# Superconductivity in compressed quasi−one-dimensional face-sharing hexagonal perovskite chalcogenides

**DOI:** 10.1126/sciadv.adv1894

**Published:** 2025-09-12

**Authors:** Feng Ke, Shanyuan Niu, Jiajia Feng, Ketao Yin, Minkyung Han, Hong Yang, Bai Yang Wang, Anna Celeste, Chunjing Jia, Bin Chen, Lin Wang, Harold Y. Hwang, Yongjun Tian, Wendy L. Mao, Yu Lin

**Affiliations:** ^1^ Stanford Institute for Materials and Energy Sciences, SLAC National Accelerator Laboratory, Menlo Park, CA 94025, USA.; ^2^ State Key Laboratory of Metastable Materials Science and Technology, Yanshan University, Qinhuangdao, Hebei 066004, China.; ^3^ College of Engineering and Applied Sciences, Nanjing University, Nanjing, Jiangsu 210093, China.; ^4^ Center for High Pressure Science and Technology Advanced Research, Shanghai 201203, China.; ^5^ School of Physics and Electronic Engineering, Linyi University, Linyi, Shandong 276005, China.; ^6^ Department of Earth and Planetary Sciences, Stanford University, Stanford, CA 94305, USA.; ^7^ Department of Applied Physics, Stanford University, Stanford, CA 94305, USA.; ^8^ Department of Physics, University of Florida, Gainesville, FL 32622, USA.

## Abstract

Oxide perovskite superconductors typically feature stacks of metal-oxygen octahedra or planar blocks connected through corners, forming three-dimensional (3D) or 2D layered structures. Here, we find a group of quasi-1D superconducting materials among hexagonal perovskite chalcogenides with face-sharing connectivity. Resistance and magnetization measurements demonstrate anisotropic superconductivity in compressed barium titanium trisulfide (BaTiS_3_) at a low hole carrier concentration of (1.6 ± 0.1) × 10^21^ per cubic centimeter, with the highest superconducting temperature (*T*_c_) reaching ~9.3 kelvin. Synchrotron x-ray diffraction indicates that the superconducting phase retains a hexagonal perovskite structure consisting of quasi-1D infinite titanium hexasulfide chains. Density functional theory calculations, combined with the observed decrease in the maximum *T*_c_ from ~9.3 to ~6.2 kelvin upon substituting sulfur with selenium, suggest that electron-phonon interactions play a key role in the pairing mechanism of superconducting BaTiX_3_ (X = sulfur and selenium). Our study offers a quasi-1D platform with face-sharing metal-chalcogen octahedra for understanding the mechanism of emerging electronic states in perovskite materials.

## Teaser

Discovery of quasi−one-dimensional hexagonal perovskite chalcogenide superconductors sheds light on superconductivity mechanisms.

## INTRODUCTION

Oxide perovskite superconductors have garnered considerable interest since the discovery of superconductivity in doped SrTiO_3_, BaPb_1-*x*_Bi*_x_*O_3_, and Ba_1-*x*_K*_x_*BiO_3_ a few decades ago (*1*–*10*). The superconducting temperature (*T*_c_) record of the oxide perovskite superconductor family has risen from hundreds of millikelvin in doped SrTiO_3_ to above the boiling temperature of liquid nitrogen in superconducting cuprates and nickelates (*11*–*19*). Typically, these materials are arranged as stacks of metal-oxygen octahedra or planar blocks connected via corner sharing to form three-dimensional (3D) or 2D layered perovskites, along with corresponding 3D or 2D electronic structures. However, many-body Hamiltonians used for exploring the superconducting mechanism, such as the Hubbard model and low-energy field theory, have no exact solution in these dimensions (*20*), posing challenges for direct comparison between experimental observations and theoretical understanding.

Metal-oxygen octahedra can also share faces to form hexagonal perovskites, creating quasi-1D octahedral chains with distinct structural connectivity. The crystal-field effect leads to different d orbital energy splitting between these two octahedral arrangements. In corner-sharing octahedra, the crystal-field effect results in triply degenerate t_2g_ (d*_xy_*, d*_yz_*, and d*_xz_*) and doubly degenerate e_g_ (d*_x²_*_−*y²*_ and d*_z²_*) orbitals, with the former typically having lower energies (*21*). In contrast, the d orbital energies in face-sharing octahedra split into a single a_1g_ orbital (similar to an atomic d*_z²_* orbital along the chain direction) and doubly degenerate $e_{g}^{\pi }$ and $e_{g}^{\sigma }$ orbitals (table S1), with the a_1g_ state usually having the lowest energy (*21*–*23*). The lowered d*_z²_* state and strong d-d orbital overlap between neighboring metal atoms enable efficient electron transport along the chain, forming a quasi-1D conductive pathway. In this dimension, theoretical models have exact solutions (*20*). Therefore, face-sharing hexagonal perovskites with quasi-1D octahedral chains may offer critical insights into the pairing mechanism of oxide perovskite superconductors. While a few oxide perovskites consisting of quasi-1D face-sharing sublattices have been studied (*24*–*27*), none have been demonstrated to exhibit superconductivity.

Recently, several superconducting material systems, including M_2_Mo_6_Se_6_ (*28*, *29*), MCr_3_As_3_ (*30*–*32*), R_3_TiTe_5_ (*33*), HfTe_3_ (*34*), and (MSe_4_)_2_I (*35*, *36*), have been found to crystallize into quasi-1D lattices. However, unlike perovskite materials, these systems lack octahedral units and octahedral connectivity, featuring much more complex crystal structures and local geometries. Despite their quasi-1D nature, their pairing mechanisms do not relate to those of oxide perovskite superconductors. In another instance, Li_0.9_Mo_6_O_17_ was widely recognized for exhibiting a 1D electronic structure with pronounced transport anisotropy, including superconductivity, yet it actually has a 3D crystal structure formed through octahedral and tetrahedral corner-sharing connectivity (*37*–*39*).

**Fig. 1.**
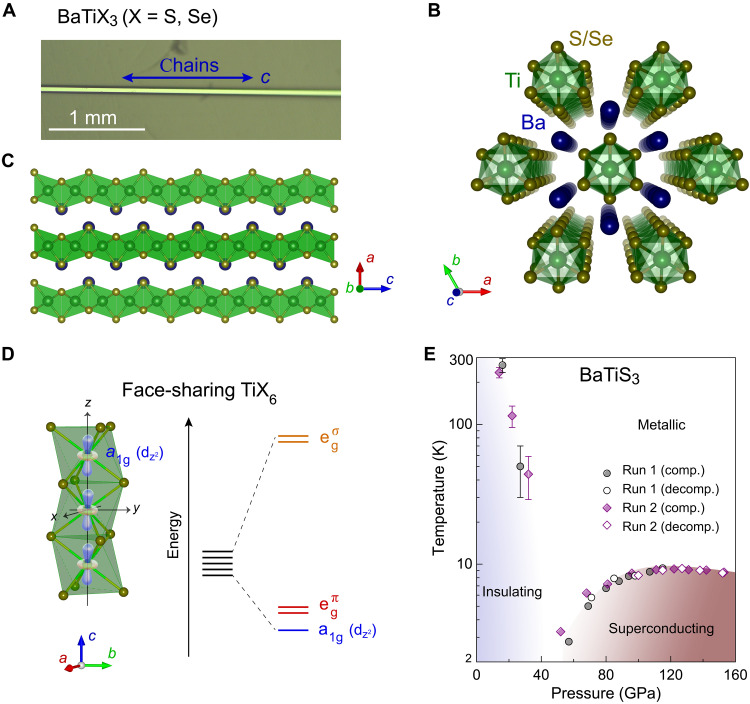
Quasi-1D structure and electronic phase diagram of BaTiX_3_ (X = S and Se) perovskite chalcogenides. (**A**) Optical image of the synthesized BaTiS_3_ needlelike single crystal. Scale bar, 1 mm. (**B** and **C**) Structural models of BaTiX_3_ showing the quasi-1D TiX_6_ octahedral chains along the *c* axis with the Ba cations residing in the cavities. Atoms in the structures are shown in blue (Ba), green (Ti), and yellow (S or Se). (**D**) Electronic configuration of face-sharing TiX_6_ octahedra. The d orbitals of Ti split into a single a_1g_ orbital and doubly degenerate $e_{g}^{\pi }$ and $e_{g}^{\sigma }\;$orbitals due to the crystal-field effect. (**E**) Electronic phase diagram of BaTiS_3_ obtained from experimental runs 1 and 2 along compression (comp.) and decompression (decomp.) cycles.

Perovskite chalcogenides represent an emerging material family that has excellent optoelectronic properties, ultralow thermal conductivity, and giant optical anisotropy (*40*–*44*). Among perovskite chalcogenides, BaTiX_3_ (X = S and Se) crystallizes into a hexagonal perovskite structure (Fig. 1, A to C), where the Ti cation is coordinated by six X anions to form TiX_6_ octahedra. The TiX_6_ octahedra in BaTiX_3_ share faces to extend into quasi-1D infinite chains along the *c* axis, with the charge-balancing Ba cation residing in the cavities of the TiX_6_ octahedral chains, offering a promising quasi-1D system for studying the emergence of intriguing electronic states and their mechanisms. The quasi-1D chain structure results in strong anisotropy in their electronic structure (*43*–*46*), where free electrons are confined to move within the infinite chains, leading to strong electron-electron correlations and a charge density wave (CDW) order (*47*). The face-sharing connectivity notably alters the orbital energies of BaTiX_3_, resulting in the a_1g_ (d*_z²_*) orbital becoming the lowest in energy that introduces orbital degrees of freedom for property modulation. In addition, the orbital energies of S-3p and Se-4p are notably higher compared to those of O-2p orbitals. Combined with the lowered Ti-d*_z²_* (a_1g_) orbital (Fig. 1D), this leads to notably smaller bandgaps, measuring only ~0.27 and ~0.20 eV in BaTiS_3_ and BaTiSe_3_ (*44*, *48*), respectively. The small bandgap can be further modulated with lattice compression. Compared to the chemical substitution method in oxide perovskites to host superconductivity, which complicates the electronic systems under study via disorder, pressure tuning allows clean access to electronic states without introducing chemical impurities, thereby simplifying the model for exploring underlying mechanisms. Collectively, these characteristics distinguish the perovskite chalcogenides from previously reported chalcogenide superconductors (table S2), including PbX (X = S and Se) (*49*–*51*), transition metal chalcogenides (*52*–*55*), iron-based chalcogenide superconductors (*56*–*58*), and other related systems (*59*–*64*), which crystallize into 3D or 2D layered structures.

**Fig. 2.**
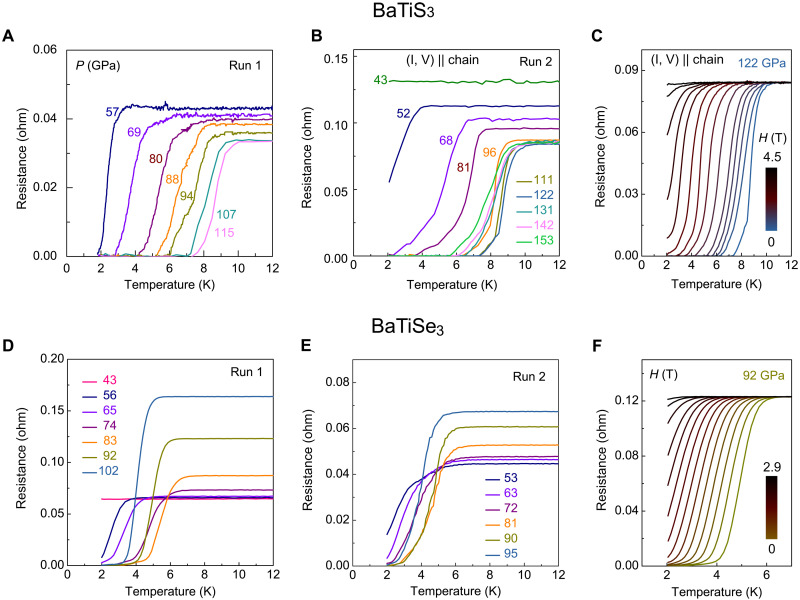
Superconducting transition of BaTiX_3_ (X = S and Se) under compression. (**A** and **B**) Resistance-temperature curves of BaTiS_3_ at representative pressures obtained from experimental runs 1 (A) and 2 (B). The data in run 2 shown in (B) were collected with the applied current and detected voltage aligned nearly along the chain [(I, V) || chain] of a BaTiS_3_ single crystal (fig. S2). (**C**) Magnetic response of the superconducting transition in BaTiS_3_ with (I, V) || chain at 122 GPa. (**D** and **E**) Resistance-temperature curves of BaTiSe_3_ single crystal at representative pressures obtained from experimental runs 1 (D) and 2 (E). (**F**) Magnetic response of the superconducting transition in BaTiSe_3_ at 92 GPa. The magnetic field was applied perpendicular to the TiX_6_ octahedral chain (*c* axis) of BaTiX_3_.

**Fig. 3.**
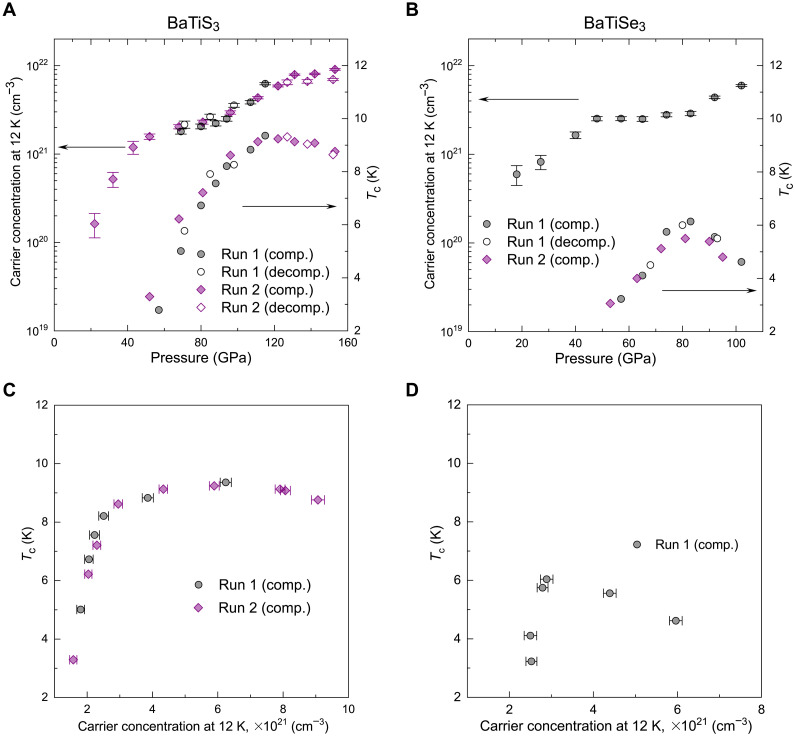
Carrier concentration (*n*) of BaTiX_3_ obtained from Hall effect measurements at 12 K under compression. (**A**) *n* of BaTiS_3_ as a function of pressure. The black circles and purple diamonds are data obtained from different comp. and decomp. cycles (runs 1 and 2), respectively. The critical temperatures (*T*_c_) for superconductivity are also plotted for comparison. (**B**) *n* of BaTiSe_3_ as a function of pressure. (**C** and **D**) *T*_c_ – *n* diagram of BaTiS_3_ (C) and BaTiSe_3_ (D) extracted from Hall effect results. The *T*_c_ values of BaTiS_3_ for run 2 shown here are those measured with (I, V) || chain, while the *T*_c_ values perpendicular to the chain are shown in fig. S2. The error bars come from the fitting of the Hall data.

**Fig. 4.**
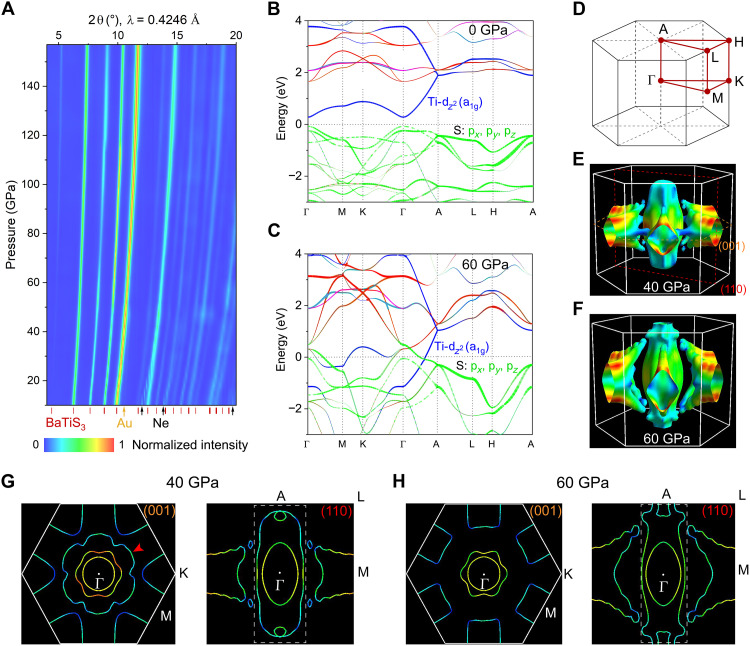
Crystal and electronic structures of superconducting BaTiS_3_. (**A**) Room-temperature XRD results of BaTiS_3_ under compression. The red tick marks correspond to the peak positions of BaTiS_3_ with the *P*6_3_/*mmc* symmetry, while the yellow and black arrows at the bottom indicate reflections associated with the gold (Au) pressure calibrant and the neon (Ne) pressure-transmitting medium, respectively. (**B** and **C**) Band structures of BaTiS_3_ at 0 GPa (B) and 60 GPa (C) calculated using *P*6_3_/*mmc* symmetry. (**D**) High-symmetry *k*-point path used for the band structure calculations. (**E** and **F**) Fermi surface of BaTiS_3_ at 40 and 60 GPa calculated using *P*6_3_/*mmc* symmetry. (**G** and **H**) Section of the Fermi surface at 40 GPa (G) and 60 GPa (H) along the (001) and (110) planes [yellow and red dashed planes shown in (E)].

In this study, we present the observation of superconductivity in quasi-1D BaTiX_3_ (X = S and Se) hexagonal perovskite chalcogenides under compression. Resistance and magnetic susceptibility measurements indicate that superconductivity occurs at an onset temperature of ~3.0 K in BaTiS_3_ and BaTiSe_3_ at ~50 GPa. With further compression, *T*_c_ increases to ~9.3 K in BaTiS_3_ and to ~6.2 K in BaTiSe_3_, respectively (Figs. 1 to 3). The measured *T*_c_ shows pronounced anisotropy parallel and perpendicular to the chains. Synchrotron x-ray diffraction (XRD) results show that the crystalline structure of the superconducting phase remains to be the quasi-1D hexagonal perovskites (Fig. 4A). Density functional theory (DFT) calculations reveal a pressure-induced Fermi surface reconstruction that favors the superconducting transition in BaTiX_3_ (Fig. 4, B to H).

## RESULTS

### Superconductivity in compressed BaTiS_3_ and BaTiSe_3_

Figure 2 and figs. S1 to S4 present the resistance-temperature curves of BaTiX_3_ (X = S and Se) at representative pressures. Three experimental runs were conducted. Robust superconducting transitions occur in BaTiS_3_ and BaTiSe_3_ above 50 GPa. With the application of a very mild pressure (i.e., <0.5 GPa), the abnormal resistance jumps that feature the CDW state at ambient pressure disappear (*47*), suggesting that the CDW state in BaTiS_3_ may have already been suppressed. BaTiS_3_ undergoes a semiconductor-to-metal transition starting at ~16 GPa and completing at ~42 GPa. At ~52 GPa, a sharp resistance drop emerges below ~3.0 K (Fig. 2B). This drop becomes increasingly pronounced at higher pressures, and eventually, a zero-resistance state appears when the sample is compressed up to ~68 GPa, indicating a superconducting transition. Upon applying a magnetic field (Fig. 2C and fig. S3), the normal state exhibits minimal magnetoresistance, but the resistance drop is progressively suppressed, consistent with the superconducting transition. Magnetization data further support the superconducting state in BaTiS_3_, where a pronounced drop occurs in the magnetic susceptibility of BaTiS_3_ at 87 GPa (fig. S5). Furthermore, the measured *T*_c_ (defined as the temperature at *R* = 0.9 *R*_0_ and *R* = 0, where *R*_0_ is the normal-state resistance at the onset of the superconducting transition) is higher with the applied current (detected voltage) parallel to the chain compared to perpendicular to the chain, indicating anisotropic superconducting behavior (fig. S2). The suppression of *T*_c_ by magnetic fields further confirms this anisotropy (fig. S3). *T*_c_ of BaTiS_3_ increases markedly by a factor of 3 with further compression up to 120 GPa, followed by a gradual reduction at higher pressures (Fig. 3A). Similarly, this dome-shaped *T*_c_ – *P* diagram is also observed in isostructural BaTiSe_3_ with the onset *T*_c_ of ~3.0 K at ~56 GPa and the maximum *T*_c_ of ~6.2 K at ~80 GPa (Figs. 2, D to F, and 3B).

### Hall effect and carrier concentration in compressed BaTiS_3_ and BaTiSe_3_

Figure 3 shows the pressure-dependent carrier concentration (*n*) of BaTiX_3_ obtained from Hall effect measurements before accessing the superconducting state (figs. S6 and S7). The main finding is that superconductivity occurs in a hole-doped range, with the onset and optimal hole-carrier concentrations for superconductivity being (1.6 ± 0.1) × 10^21^ and (6.2 ± 0.13) × 10^21^ for BaTiS_3_ and (2.5 ± 0.1) × 10^21^ and (2.9 ± 0.15) × 10^21^ cm^−3^ for BaTiSe_3_, respectively. The Hall effect results show that *n* of BaTiS_3_ at 12 K, i.e., *n*(12 K), increases by approximately one order of magnitude following the pressure-induced metallization and reaches (1.6 ± 0.1) × 10^21^ cm^−3^ at ~52 GPa where the superconducting transition occurs at low temperature. With further compression, *n*(12 K) increases smoothly with *T*_c_ and is (6.2 ± 0.13) × 10^21^ cm^−3^ at ~115 GPa where *T*_c_ achieves its maximum. *T*_c_ reduces smoothly at higher pressures, while *n*(12 K) continues the rising trend. The dome-shaped *T*_c_ – *n* diagram (Fig. 3C) resembles the *T*_c_ evolution from under-doped to over-doped compositions seen in superconducting cuprates (*11*). In the case of BaTiSe_3_, *n*(12 K) evolves in a similar fashion to BaTiS_3_ (Fig. 3, B and D). The onset *n* of BaTiX_3_ for superconductivity is comparable to that of BaPb_1-*x*_Bi*_x_*O_3_ oxide perovskite where unexpectedly high *T*_c_ values were observed at rather low carrier densities (*5*).

### Structure of superconducting BaTiS_3_ and BaTiSe_3_

The crystal structure of BaTiX_3_ under compression was determined by synchrotron XRD measurements on powdered samples (Fig. 4A and figs. S8 to S11), combined with first-principles calculations (fig. S12). XRD results show that no additional peaks appear in BaTiS_3_ up to 157 GPa and BaTiSe_3_ up to 74 GPa, indicating their structural stability under compression. Rietveld fitting suggests that all the XRD patterns can be well indexed to the *P*6_3_*cm*, *P*6_3_*mc*, and *P*6_3_/*mmc* hexagonal perovskite structures with quasi-1D TiX_6_ octahedral chains (figs. S10 and S11). Enthalpy calculations (fig. S12) reveal that the *P*6_3_/*mmc* structure has the lowest enthalpy compared to the *P*6_3_*cm* and *P*6_3_*mc* structures at above 60 GPa, implying that the superconducting phase has a *P*6_3_/*mmc* structure. More discussion about the potential phase change can be found in fig. S12. When superconductivity emerges in BaTiS_3_ and BaTiSe_3_ (fig. S13), the unit cell volume reduces by 18.7 and 24.7%, respectively, compared to the ambient value. By fitting the pressure-dependent unit cell volume using a third-order Birch-Murnaghan equation of state, a bulk modulus of *B*_0_ = 55.4 ± 2.9 GPa (*B*_0_′ = 4.1 ± 0.3) and *B*_0_ = 59.5 ± 3.4 GPa (*B*_0_′ = 4.3 ± 0.4) is obtained for BaTiS_3_ and BaTiSe_3_, respectively (fig. S13), indicating that hexagonal perovskite chalcogenides have softer lattices compared to the parent compounds of related superconducting oxide perovskites, such as BaTiO_3_ (*B*_0_ = 139.2 GPa) and SrTiO_3_ (*B*_0_ = 178.8 GPa) (*65*).

### Electronic structure of superconducting BaTiS_3_ and BaTiSe_3_

We conducted DFT calculations on BaTiS_3_ to study the electronic structure and phonon dispersion of the superconducting phase using the *P*6_3_/*mmc* space group (figs. S14 to S17) and found clear reconstruction in the Fermi surface that favors the emergence of superconductivity. The ambient-pressure band structure of BaTiS_3_ exhibits clear quasi-1D characteristics (Fig. 4B). The valence band maximum and conduction band minimum are mainly from the S-3p and Ti-3d*_z²_* (a_1g_) states. The Ti-3d*_z²_* (a_1g_) conduction band expands substantially approaching the Fermi surface along the Γ-A path (the chain direction; *c* axis) and has a small direct bandgap of ~0.25 eV with the S-3p valence band at the Γ zone center, consistent with the experimental value (*44*). In addition, the Ti-3d bands are flat along the A-L-H path (perpendicular to the TiS_6_ octahedral chains), indicating that free electrons move along the quasi-1D TiS_6_ octahedral chains but are restricted from moving between neighboring chains. Ba^2+^ ions have negligible contributions to the valence and conduction bands near the Fermi surface and are not involved in the conduction process (fig. S14), further supporting that the conducting quasi-1D TiS_6_ chains are well separated by the Ba^2+^ cations. With the application of pressure, the Ti-3d*_z²_* (a_1g_) and S-3p orbitals broaden markedly and cross the Fermi surface (fig. S15), resulting in bandgap closure, consistent with the semiconducting-to-metallic transition observed in our resistance-temperature data. Several hole pockets form after the crossing of the S-3p orbitals over the Fermi surface, generating hole carriers, which agrees with the Hall effect results. All these changes suggest notable reconstruction of the Fermi surface of BaTiS_3_. The Fermi surface calculations indicate that the Fermi surface topology is considerably different at 40 and 60 GPa, which are below and above the superconducting transition pressure in BaTiS_3_, respectively. The Fermi surface is primarily composed of the Ti-3d*_z²_* (a_1g_) and S-3p orbitals, indicating their relevance in the superconducting behavior. The outer pocket around the Γ point (red arrow in Fig. 4G) merges with the six pockets around the K points after the superconducting transition. The second elliptical pocket around the Γ point [shown in white rectangles in Fig. 4 (G and H)] expands markedly toward the A point, forming a typical metallic open Fermi surface extending over almost the entire Brillouin zone, predominantly contributing to carrier transport along the chain direction. This Fermi surface reconstruction increases the density of states at the Fermi level [*N*(*E*_F_)], a dominant parameter in the occurrence of superconductivity.

## DISCUSSION

Our study reveals that electron-phonon interactions play a substantial role in the superconductivity of BaTiX_3_. Band structure and Fermi surface calculations show that the Ti-3d*_z²_* (a_1g_) and S-3p orbitals contribute most to the Fermi surface of superconducting BaTiS_3_. Pressure-induced crossover of the S-3p orbitals to the Fermi surface generates hole carriers, favoring the superconducting transition, possibly via electron-phonon coupling. This is evidenced by the reduction of the maximum *T*_c_ from ~9.3 to ~6.2 K when S is substituted with the heavier Se. According to the Bardeen-Cooper-Schrieffer theory, *T*_c_ ∝ *M*^−α^, where *M* and α are the isotopic atom mass and isotopic coefficient, respectively. Typically, a large value of α (>0.3) indicates electron-phonon coupling (*6*). Assuming that replacing S with Se is analogous to the isotope effect, the relationship *T*_c_(S)/*T*_c_(Se) = [*M*(Se)/*M*(S)]^α^ suggests an α value of 0.45, indicating that electron-phonon interactions contribute considerably to the superconductivity of BaTiX_3_. In addition, the Ti-3d electrons show strong electron-electron correlations that require an effective Hubbard *U* value of 3 eV to describe this correlation in electronic structure calculations including *N*(*E*_F_), band structure, and Fermi surface, underscoring its potential role in facilitating the superconducting behavior of BaTiX_3_. The contribution of electron-electron correlations in the Cooper pairing has been previously demonstrated in BaPb_1-*x*_Bi*_x_*O_3_ and Ba_1-*x*_K*_x_*BiO_3_ (*8*, *9*, *66*). The electronic phase diagrams of BaTiX_3_ are similar to those of BaPb_1-*x*_Bi*_x_*O_3_ and Ba_1-*x*_K*_x_*BiO_3_, where superconductivity emerges at rather low carrier densities (*5*), accompanied by the suppression of the CDW order. A systematic study of the superconducting mechanism in BaTiX_3_ could provide valuable insights into understanding the puzzling pairing mechanism in BaPb_1-*x*_Bi*_x_*O_3_ and Ba_1-*x*_K*_x_*BiO_3_ oxide perovskite superconductors.

In summary, our study finds superconductivity in quasi-1D hexagonal perovskite chalcogenides with face-sharing connectivity. The face-sharing connectivity notably alters orbital energies and electronic structures, as evidenced by the markedly distinct band structures and Fermi surface of superconducting BaTiX_3_ compared to those of corner-shared oxide perovskite superconductors (*11*). Unlike cuprate superconductors, where the planar d*_x²_*_−*y²*_ state contributes to superconductivity, the crystal-field effect arising from face-sharing octahedra in BaTiX_3_ causes the Ti-3d*_z²_* (a_1g_) orbital to have the lowest energy. This facilitates electron hopping along the chain, forming a quasi-1D conductive channel and resulting in highly anisotropic superconductivity. These features make BaTiX_3_ an exceptional quasi-1D platform for examining and refining theories of correlated electronic states. Furthermore, superconductivity in quasi-1D BaTiS_3_ and BaTiSe_3_ emerges at low hole carrier concentrations of (1.6 ± 0.1) × 10^21^ and (2.5 ± 0.1) × 10^21^ cm^−3^, respectively, which can be achieved through chemical substitution. This points to the promising potential for synthesizing a group of quasi-1D face-sharing perovskite chalcogenide superconductors that can be stable at (near) ambient pressure.

## MATERIALS AND METHODS

### Material syntheses

BaTiX_3_ (X = S and Se) single crystals were grown via a chemical vapor transport method (*44*). The stoichiometric mixture of binary chalcogenide (barium chalcogenide), titanium, and sulfur precursors was loaded into a sealed quartz ampoule along with iodine pieces in a nitrogen-filled glove box. The precursors were then heated up to 1050°C at a rate of 100°C/hour, and the temperature was held for 100 hours before cooling down to 950°C at a rate of 10°C/hour. The products were then cooled to room temperature by turning off the power of the furnace. As-grown BaTiX_3_ single crystals were collected under a microscope and stored in a nitrogen-filled glove box.

### Resistance measurements

Resistance measurements on BaTiX_3_ (X = S and Se) single crystal samples at high pressure up to 153 GPa were conducted using a nonmagnetic diamond anvil cell made from Be-Cu alloy. Beveled diamond anvils with inner/outer diamond culets measuring 100/300 or 150/300 μm in diameter were used. An insulating gasket was fabricated from a preindented rhenium sheet entirely enveloped by a compact insulating layer made of a mixture of epoxy and cubic boron nitride (1:10 weight ratio). The detailed preparation process of an insulating gasket can be found in our previous studies (*61*, *67*, *68*). A BaTiX_3_ single crystal sample measuring 30 μm by 30 μm by 10 μm [with the chain direction (*c* axis) lying within the plane of the diamond culet surface; figs. S1 and S2] along with NaCl fine powder serving as the pressure-transmitting medium was loaded for resistance measurements. Four platinum (Pt) foil electrodes were prepared by a hand-wiring method. The assembled Be-Cu cell was subsequently loaded into a Physical Property Measurement System (Quantum Design, PPMS-9, and DynaCool) for measurements at low temperatures (1.8 to 300 K) and under high magnetic fields (−9 to 9 T). Hall measurements were performed with the applied magnetic field perpendicular to the *c* axis of the BaTiX_3_ single crystal. To examine the electrical transport anisotropy of BaTiS_3_, the single crystal sample in experimental run 2 was carefully aligned with the probing electrodes, ensuring that the current (voltage) was applied (detected) nearly parallel and perpendicular to the chain direction (*c* axis) of BaTiS_3_ (fig. S2). Pressure was calibrated at room temperature using the diamond Raman peak (~1333 cm^−1^ at ambient pressure) (*69*). Pressure differences of a few gigapascals (<5%) were observed between the center and edge of the sample, for example, ~3 GPa at ~75 GPa and ~7 GPa at ~153 GPa (fig. S18). Pressure values used for plotting the data in the figures correspond to those measured at the center of the sample.

### Magnetic susceptibility measurements

Magnetic susceptibility measurements were conducted using a Magnetic Property Measurement System (Quantum Design, MPMS3) with a superconducting quantum interference device. A homemade Be-Cu cell (~8 mm in diameter) with diamond culets of 200 μm in diameter was used. A single crystal sample measuring ~120 μm in diameter and ~14 μm in thickness was loaded for experiments. No pressure-transmitting medium was loaded to avoid any unintended influence of the pressure medium on the magnetic signals. A magnetic field of 50 Oe was applied for the temperature-dependent magnetic susceptibility measurements. Before taking data on the sample, the empty diamond anvil cell made from Be-Cu alloy with a Be-Cu alloy gasket was loaded into the system for background collection. A sample of single-crystal BaTiS_3_ was then loaded into the cell for high-pressure experiments up to 90 GPa. Two sets of measurements were conducted: one for the field-cooled (*V*_FC_) measurement and the other for the zero−field-cooled measurement (*V*_ZFC_). The magnetic susceptibility data were then calculated as *V*_SC_ = *V*_ZFC_ − *V*_FC_.

### Synchrotron XRD measurements

Synchrotron XRD measurements at high pressure were conducted at sector 16 of the Advanced Photon Source (APS), Argonne National Laboratory (ANL), and beamline 12.2.2 of the Advanced Light Source (ALS), Lawrence Berkeley National Laboratory, with x-ray wavelengths of 0.4246 and 0.4959 Å, respectively. Beveled Boehler-type diamond anvils with an inner/outer culet diameter of 100/300 or 150/300 μm and a tungsten gasket were used to generate pressures above 157 GPa. The sample chamber was prepared by preindenting a tungsten gasket to ~30 μm in thickness and drilling a hole with a diameter of 30 to 50 μm in the preindented area. Powdered samples, obtained by grinding several pieces of single-crystal BaTiX_3_ in a nitrogen-filled glove box for half an hour, were loaded into the sample chamber along with a small piece of compacted gold (Au) powder as a pressure calibrant. Neon (Ne) was loaded as the pressure-transmitting medium using a gas-loading system for BaTiS_3_ up to 157 GPa. No pressure medium or silicone oil was used for the XRD measurements of BaTiSe_3_.

### DFT calculations

DFT calculations including structural optimization, band structures, and Fermi surface were carried out using the Perdew-Burke-Ernzerhof exchange-correlation functional of the generalized gradient approximation implemented in the Vienna ab initio simulation package (*70*, *71*). A plane-wave energy of 700 eV was set for the projector-augmented wave pseudopotentials. The valence electrons of Ba, Ti, and S atoms were 5s^2^5p^6^6s^2^, 3p^6^4s^2^3d^2^, and 3s^2^3p^4^, respectively. A DFT + *U* method with different effective Hubbard *U* values (*U*_eff_ = *U* – *J*) was adopted for Ti to correct the self-interaction error inherent in the pure DFT model for transition metals with tightly localized d electrons (*72*). The obtained bandgap using a *U*_eff_ value of 3.0 eV agrees well with the experimental value of BaTiS_3_ at ambient pressure. A fine Monkhorst-Pack Brillouin zone sampling grid with a resolution of 0.03 × 2π Å^−1^ was applied in our calculations. Both atomic positions and lattice parameters were relaxed until all the forces of the ions were smaller than 10^−3^ eV/Å. The input structures of BaTiS_3_ at ambient pressure were from the Inorganic Crystal Structure Database (ICSD-18201) and previous studies (*44*, *47*). For the calculations at high pressure, we input the lattice parameters obtained from the refinements of XRD results and then performed structural optimization including the lattice parameters and atomic positions.

## Supplementary Materials

### This PDF file includes:

Figs. S1 to S18 Tables S1 to S3 References
