# Supplementary material for: Superconductivity in compressed quasi−one-dimensional face-sharing hexagonal perovskite chalcogenides
Source: Sci Adv. 2025 Sep 12;11(37):eadv1894. doi: 10.1126/sciadv.adv1894 (PMC12429050; doi:10.1126/sciadv.adv1894)
Supplement: Supplementary file 1 — Figs. S1 to S18 Tables S1 to S3 References [file sciadv.adv1894_sm.pdf]

Science Advances
AAAS

Supplementary Materials for

Superconductivity in compressed quasi−one-dimensional face-sharing hexagonal perovskite chalcogenides

Feng Ke *et al.*

Corresponding author: Wendy L. Mao, wmao@stanford.edu; Yu Lin, lyforest@slac.stanford.edu

Sci. Adv. **11**, eadv1894 (2025)
DOI: 10.1126/sciadv.adv1894

This PDF file includes:

Figs. S1 to S18
Tables S1 to S3
References

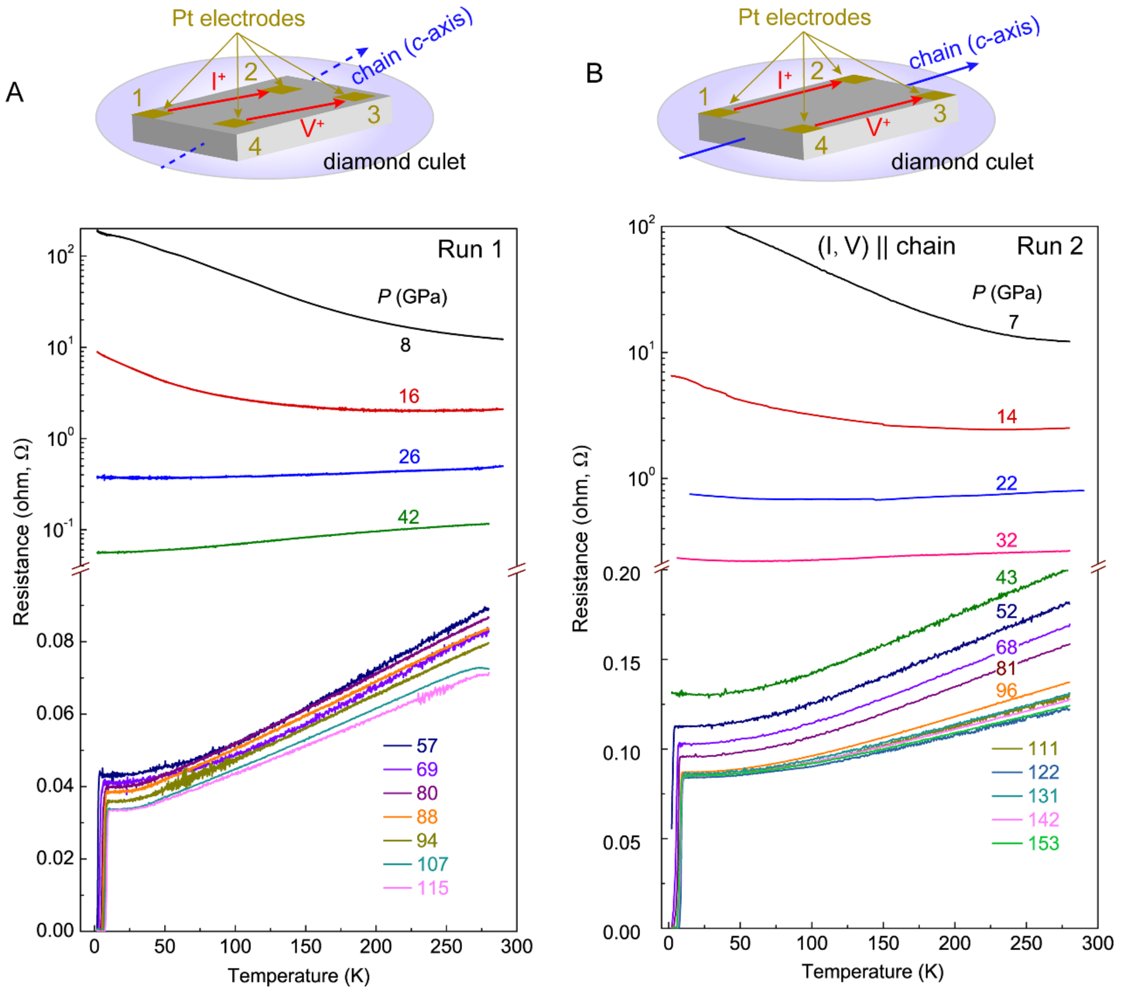

Fig. S1. Resistance – temperature (R – T) curves of $BaTiS_3$ within 2 – 300 K range, obtained from experimental runs 1 (A) and 2 (B). In run 1, the $BaTiS_3$ single crystal was loaded with the chain (*c*-axis) within the plane of the diamond culet, but the crystal's orientation with respect to the current and voltage was not defined. In run 2, the crystal was carefully aligned with the probing electrodes to ensure that the applied current and detected voltage were nearly parallel or perpendicular to the chain (*c*-axis) of $BaTiS_3$. This alignment enables the study of electrical transport anisotropy including superconductivity. The data shown in (B) are the R – T curves with the applied current nearly parallel to the chain direction.

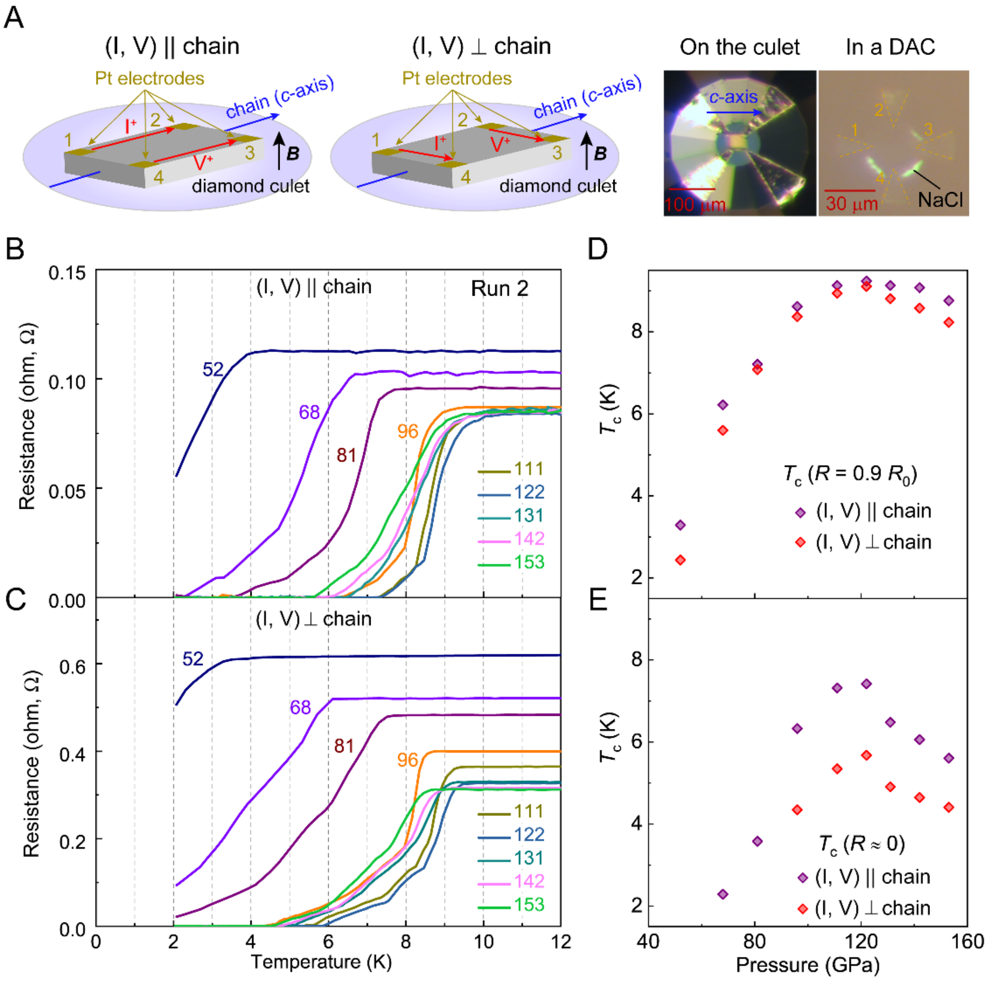

Fig. S2. R – T curves within 2 – 12 K range with the applied current and detected voltage nearly parallel and perpendicular to the chain and the T_c – P diagrams of $BaTiS_3$ obtained from experimental run 2. A, Electrode configuration and experimental micrographs of a single crystal sample loaded on the culet and in a diamond anvil cell (DAC) for resistance measurements with the applied current and detected voltage nearly parallel ((I, V) || chain) and perpendicular ((I, V) ⊥ chain) to the chain. **B** and **C**, R – T curves measured with (I, V) || chain (B) and (I, V) ⊥ chain (C), respectively. **D** and **E**, T_c – P diagrams of $BaTiS_3$. T_c is defined as the temperature corresponding to R = 0.9 R_0 (D) and R ≈ 0 (E), respectively, where R_0 is the normal-state resistance at the onset of the superconducting transition. The resistance and T_c (R ≈ 0) measured parallel and perpendicular to the chain exhibit clear anisotropy.

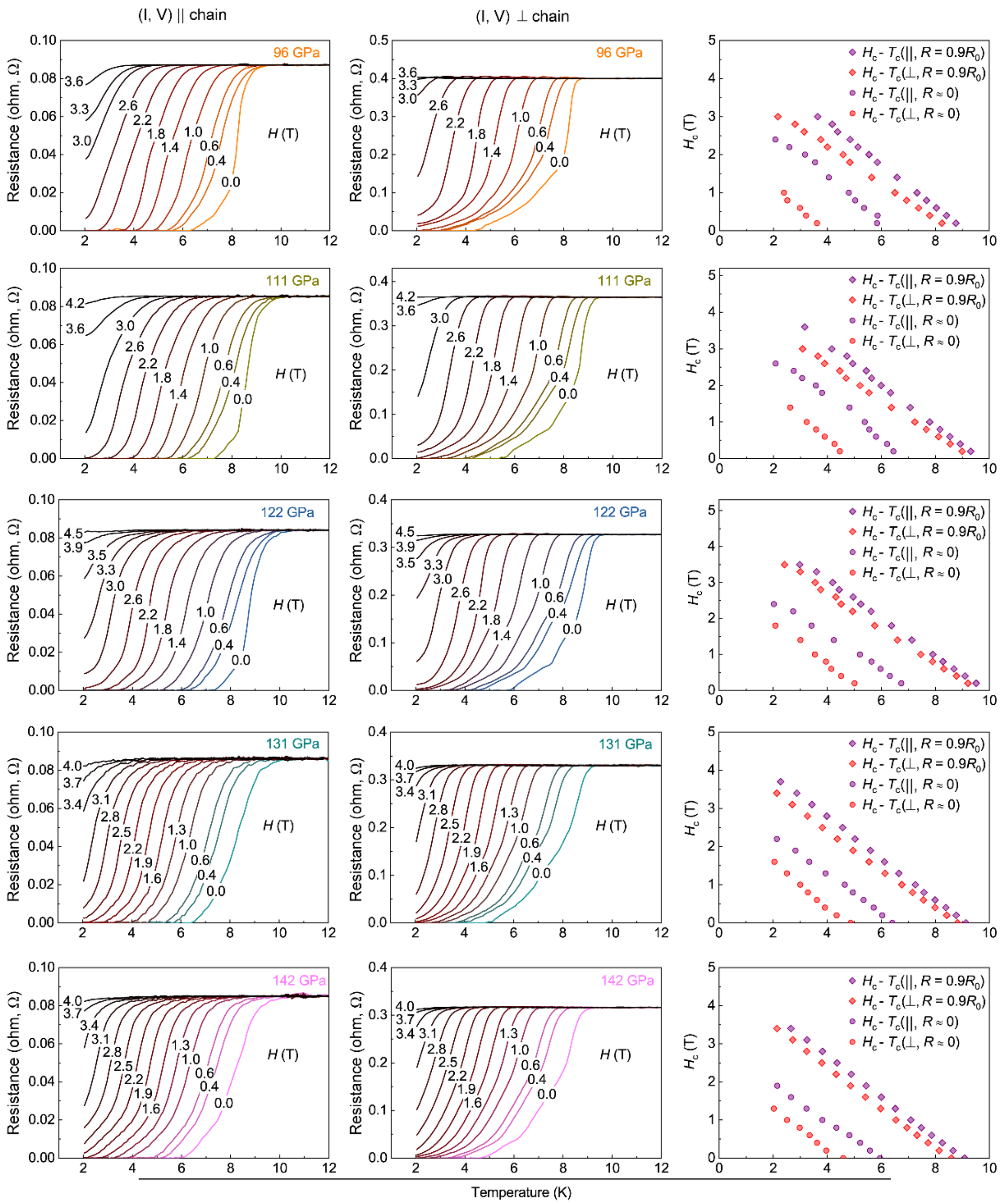

Fig. S3. Magnetic-field response of superconducting $BaTiS_3$ at representative pressures. The data were measured with the applied current and detected voltage aligned nearly parallel ((I, V) || chain) and perpendicular ((I, V) ⊥ chain) to the chain. The applied magnetic field is perpendicular to the chain (fig. S2). The temperature dependent critical magnetic field ($H_c – T_c$) curves show clear anisotropy. The applied current and current density were 0.2 mA and ~0.83 * 10^3 mA/mm^2 (the section area of the sample is ~8 μm in thickness and ~30 μm in length), respectively.

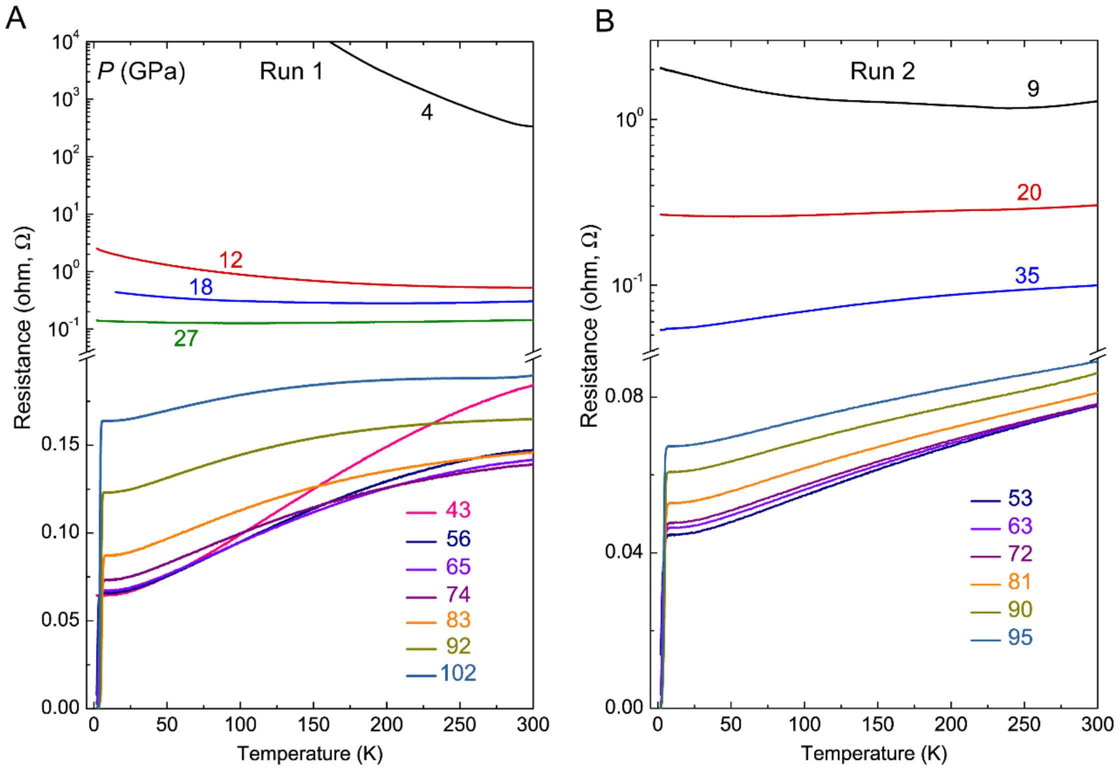

Fig. S4. *R* – *T* curves of $BaTiSe_3$ within 2 – 300 K range obtained from experimental runs 1 and 2. The $BaTiSe_3$ single crystal was loaded with the chain (*c*-axis) aligned nearly parallel to the diamond culet, but the crystal's orientation with respect to the applied current and detected voltage was not defined.

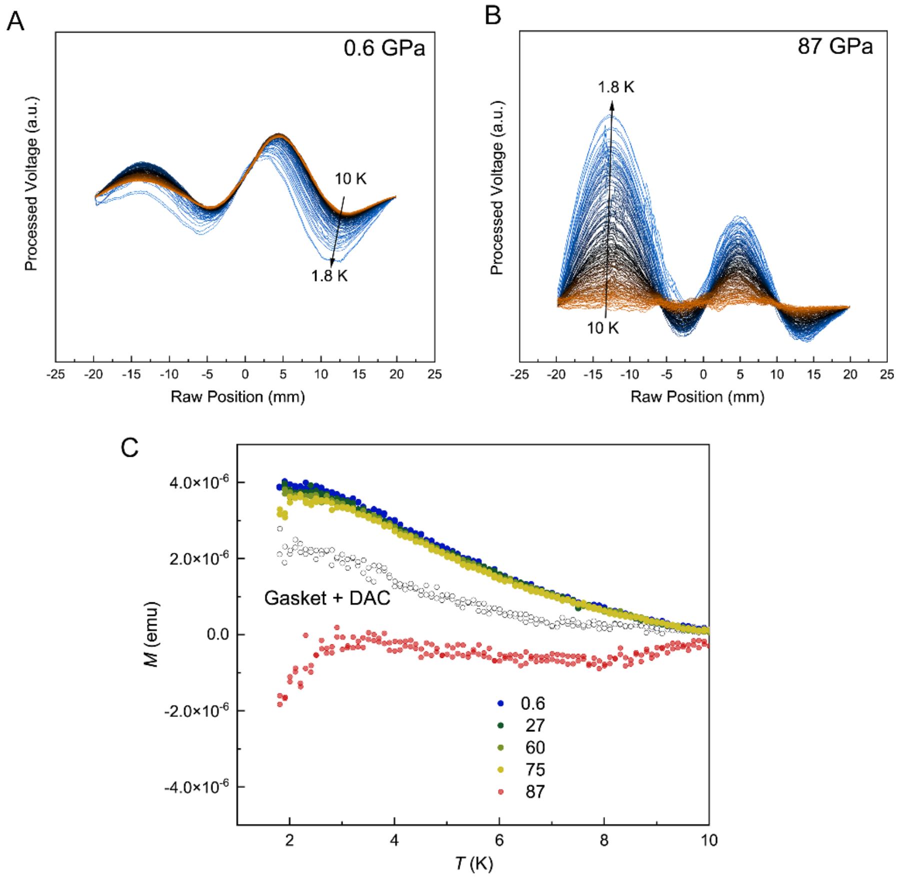

Fig. S5. Magnetic susceptibility (*M*) of $BaTiS_3$ at representative pressures. A and **B,** Raw magnetic susceptibility measurement data from 1.8 to 10.0 K with a step size of 0.1 K at 0.6 and 87 GPa conducted in MPMS. **C**, Magnetic susceptibility data calculated with $V_{SC} = V_{ZFC} - V_{FC}$. The background signal from the gasket and the DAC is shown in open black circles. A magnetic field of 50 Oe was applied for the $M - T$ measurements. The size of the $BaTiS_3$ sample at ~87 GPa was ~120 μm in diameter and ~8 μm in thickness. Based on the sample geometry, the demagnetizing factor was estimated to be ~0.87 (Calculator for demagnetizing factor, http://www.magpar.net /static/magpar/doc/html/demagcalc.html). The diamagnetic ratio can be estimated: $\chi = \frac{4\pi M}{H-4\pi Mn}$, where M, H, and n is the magnetization in unit volume, external magnetic field, and demagnetizing factor, respectively. We obtained $\chi \approx$ -0.95, that is, the superconducting volume of $BaTiS_3$ at 87 GPa is ~95%. We noticed that the onset

pressure and T_c are slightly different from those observed from the $R - T$ curves, which may arise from the varying sensitivities of different methods used for superconductivity measurements, as well as from pressure gradients within a DAC. Generally, a superconducting transition can be identified from magnetic susceptibility measurements when the material enters a zero-resistance state. That being said, the T_c(onset) obtained from the $M - T$ curves is usually comparable to the $T_c(R \approx 0)$, which is smaller than the $T_c(R = 0.9\ R_0)$ and T_c(onset) measured from the $R - T$ curves. Additionally, pressure gradients may make non-negligible contributions to the observed difference in T_c, since they may significantly broaden the superconducting transition temperature.

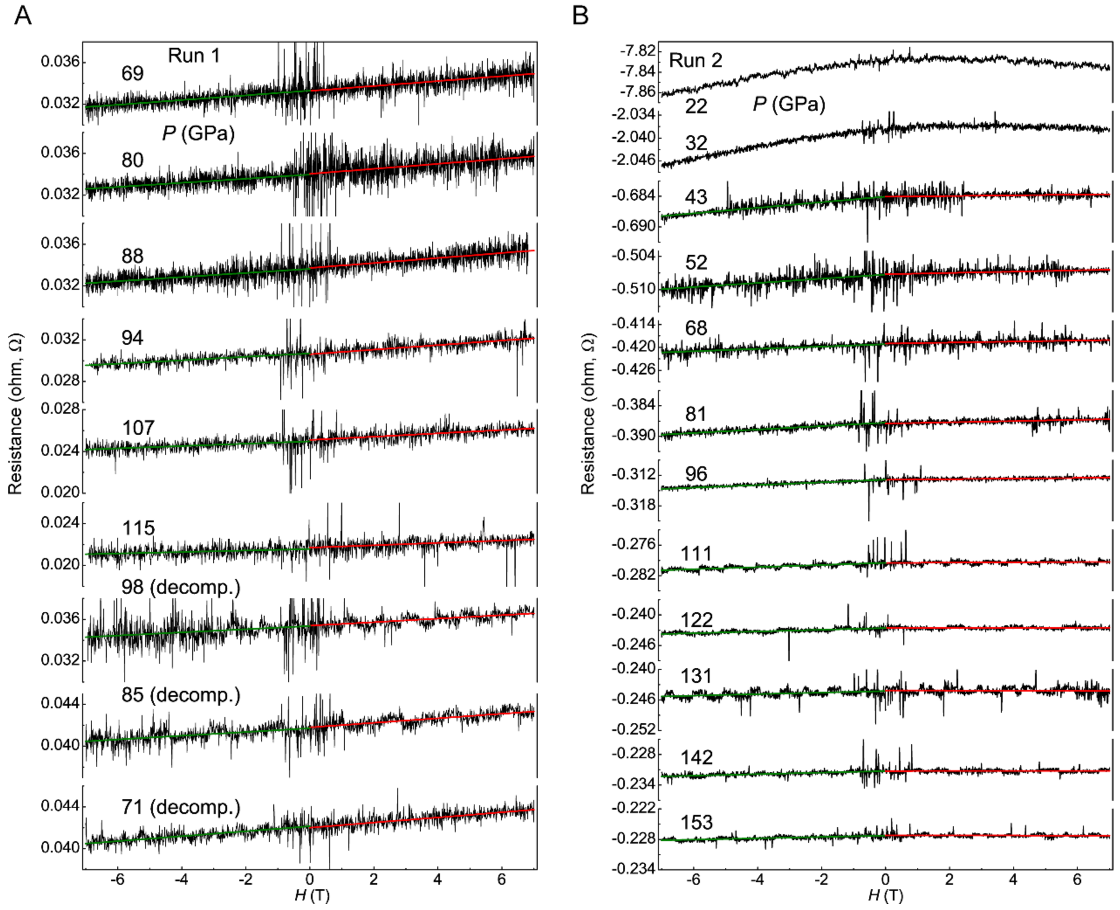

Fig. S6. Raw data of Hall effect measurements on $BaTiS_3$ under compression. **A** and **B**, Raw data obtained from the experimental runs 1 (A) and 2 (B) of $BaTiS_3$, respectively. Magnetic fields (-7 – 7 T) were applied perpendicular to the *c*-axis of the $BaTiS_3$ single crystal (Fig. S2). The green and red lines are the linear fits of the data from -7 to 0 T and 0 to 7 T, respectively. Hall resistances were then obtained by subtracting the interpolated resistance values at negative magnetic fields from those at related positive magnetic fields to exclude the asymmetric effect. By analyzing the Hall data, positive Hall coefficients were obtained, indicating that $BaTiS_3$ hosts hole-type carriers under compression.

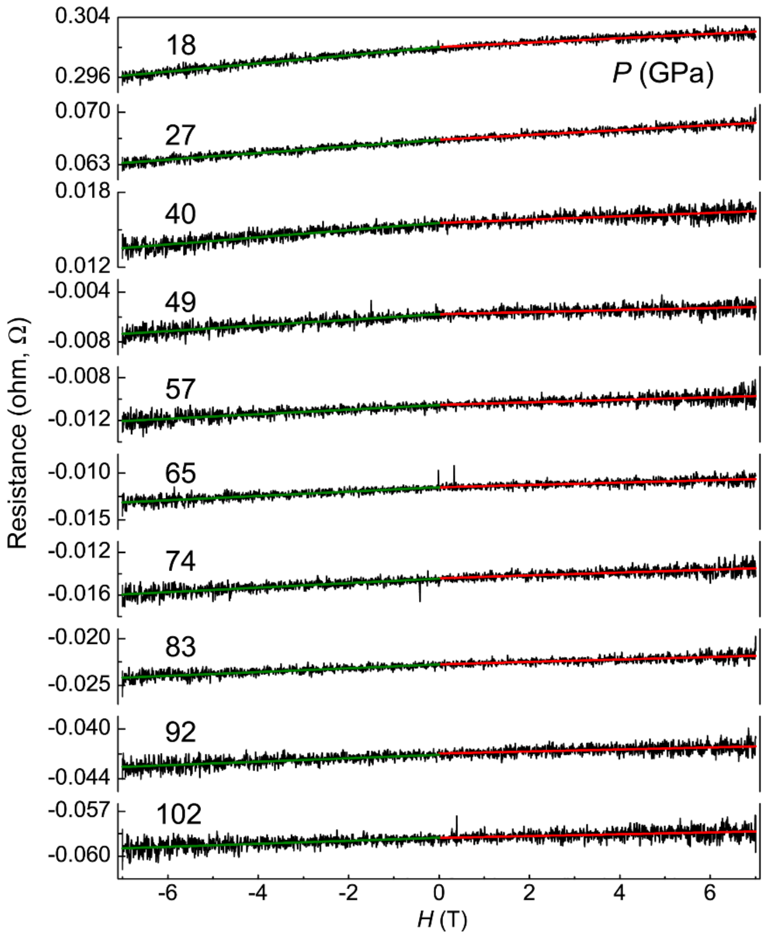

Fig. S7. Raw data of Hall effect measurements on $BaTiSe_3$ under compression. Magnetic fields (-7 – 7 T) were applied perpendicular to the *c*-axis of the $BaTiSe_3$ single crystal. The green and red lines are the linear fits of the data from -7 to 0 T and 0 to 7 T, respectively. Hall resistances were then obtained by subtracting the interpolated resistance values at negative magnetic fields from those at related positive magnetic fields to exclude the asymmetric effect. By analyzing the Hall data, positive Hall coefficients were obtained, indicating that $BaTiSe_3$ hosts hole-type carriers under compression.

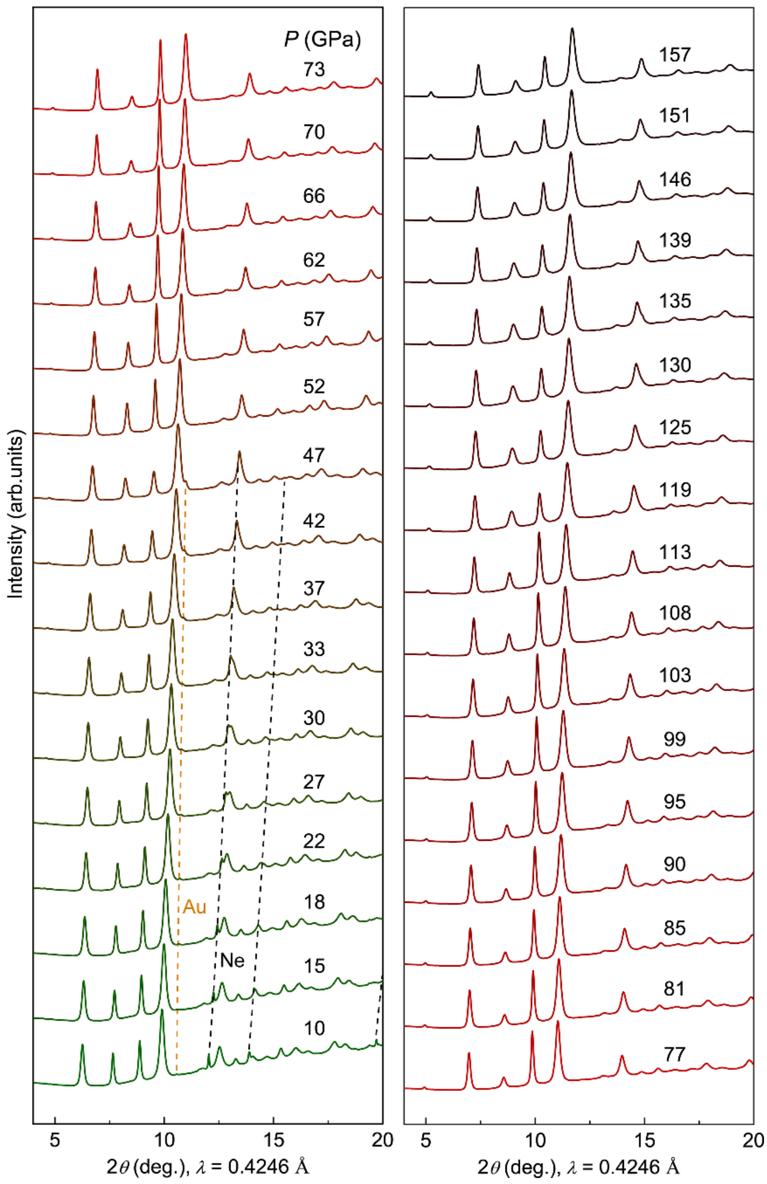

Fig. S8. XRD patterns of $BaTiS_3$ under compression. Neon (Ne) was loaded as the pressure transmitting medium. Gold (Au) and a ruby ball are used to calibrate the pressure. The yellow dashed line in the left panel is the weak signal from the Au pressure calibrator. The black dashed lines are the peaks from the Ne pressure medium. The slight changes in the relative peak intensities may result from the preferred orientation of the samples as pressure increases.

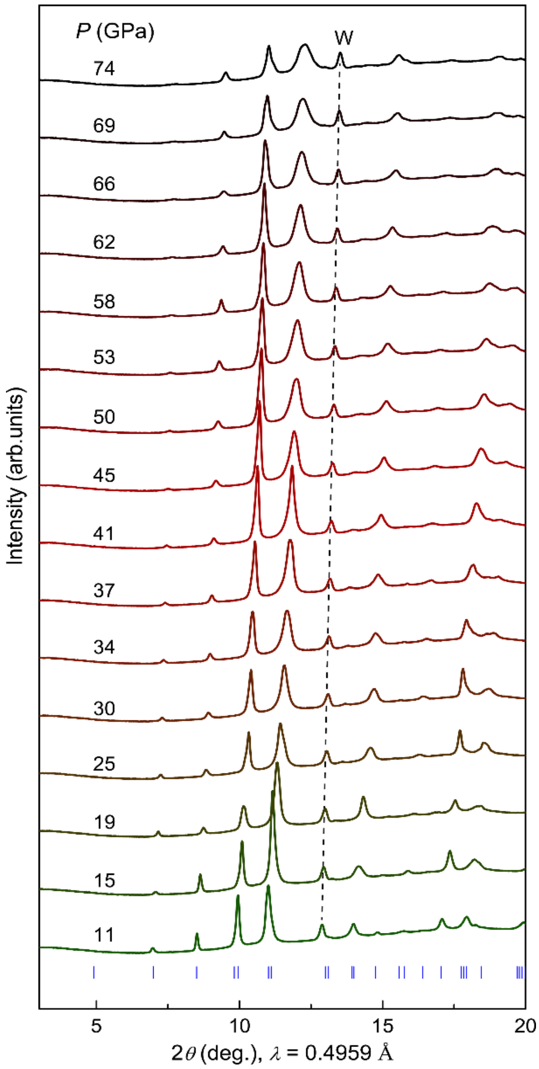

Fig. S9. XRD patterns of $BaTiSe_3$ under compression. Silicone oil was loaded as the pressure transmitting medium. Gold (Au) and a ruby ball are used to calibrate the pressure. The black dashed line represents a peak from the tungsten (W) gasket used in our experiments. The blue vertical tick marks indicate the indexed peaks corresponding to the $P6_3mc$ structure. The most intense peak at ~11.5° at low pressure consists of two reflections, which gradually separate, leading to peak broadening with increasing pressure (fig. S10). Pressure conditions provided by the silicone oil pressure medium also broaden the reflection peaks at higher pressures.

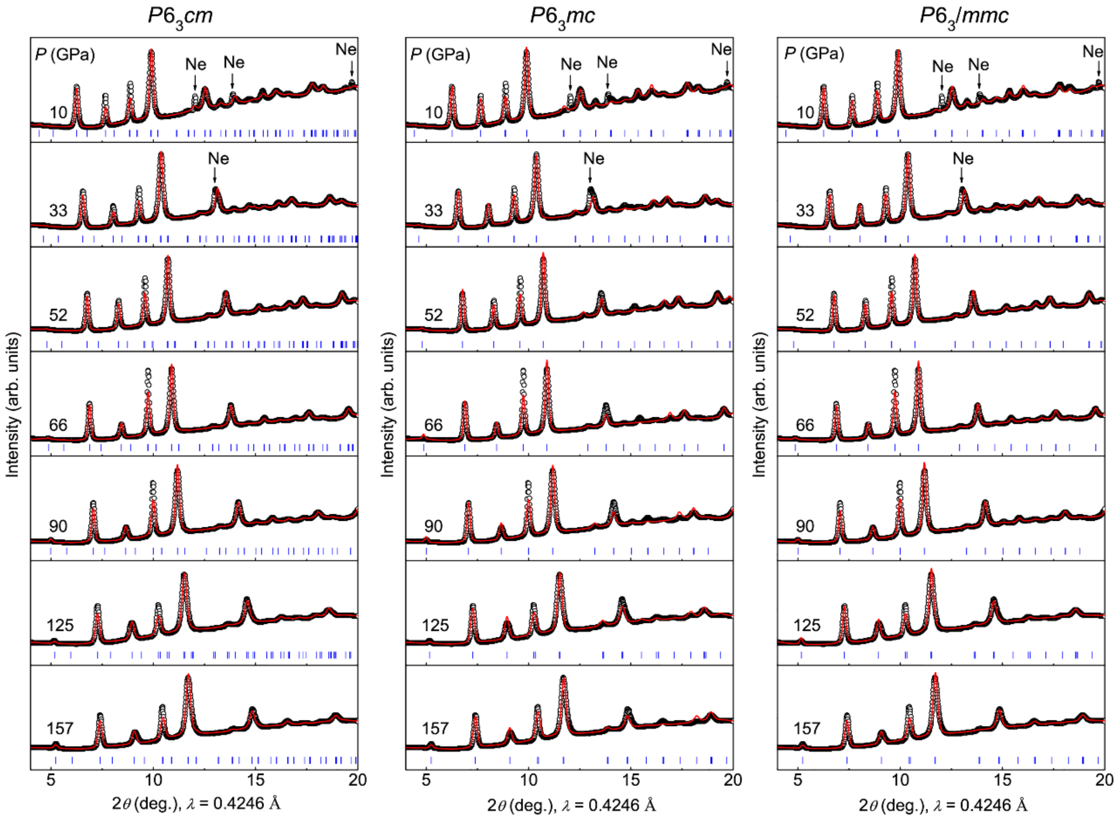

Fig. S10. Rietveld fitting results of the XRD patterns of $BaTiS_3$ under compression. The open circles and red lines are the experimental data and fitting results using $P6_3cm$, $P6_3mc$ and $P6_3/mmc$ symmetries. The blue vertical tick marks indicate the indexed peaks corresponding to the related structure. The arrows mark the peaks from the Ne pressure medium used in our XRD experiments. All the XRD patterns can be well indexed into these three hexagonal perovskite structures with quasi-1D TiX_6 octahedral chains. These structures are very similar, with only slight differences in their atomic positions, which are beyond the resolution of XRD to distinguish and resolve the exact crystal structure. Enthalpy calculations (fig. S12) indicate that the $P6_3/mmc$ structure has the lowest enthalpy compared to the $P6_3cm$ and $P6_3mc$ structures above 60 GPa, suggesting that the superconducting phase of $BaTiS_3$ has the $P6_3/mmc$ structure. A recent study reported that the calculated peaks based on the $P6_3/mmc$ structure showed a slight mismatch with their experimental results, and they indexed the high-pressure phase above 10 GPa to the $Cmc2_1$ structure (*73*). Our Rietveld refinements did not

observe such a mismatch, and the calculated peaks of the $P6_3/mmc$ structure match well with our experimental XRD patterns. This difference may be caused by the different pressure-transmitting media used in the previous study (silicone oil) and our study (neon) for XRD measurements. Neon can provide a better quasi-hydrostatic pressure environment, resulting in a lower pressure gradient compared to silicone oil (*74*). The large pressure gradients introduced by silicone oil leads to significant broadening and shifting of diffraction peaks, which subsequently contributes to the mismatch between the calculation and experimental results.

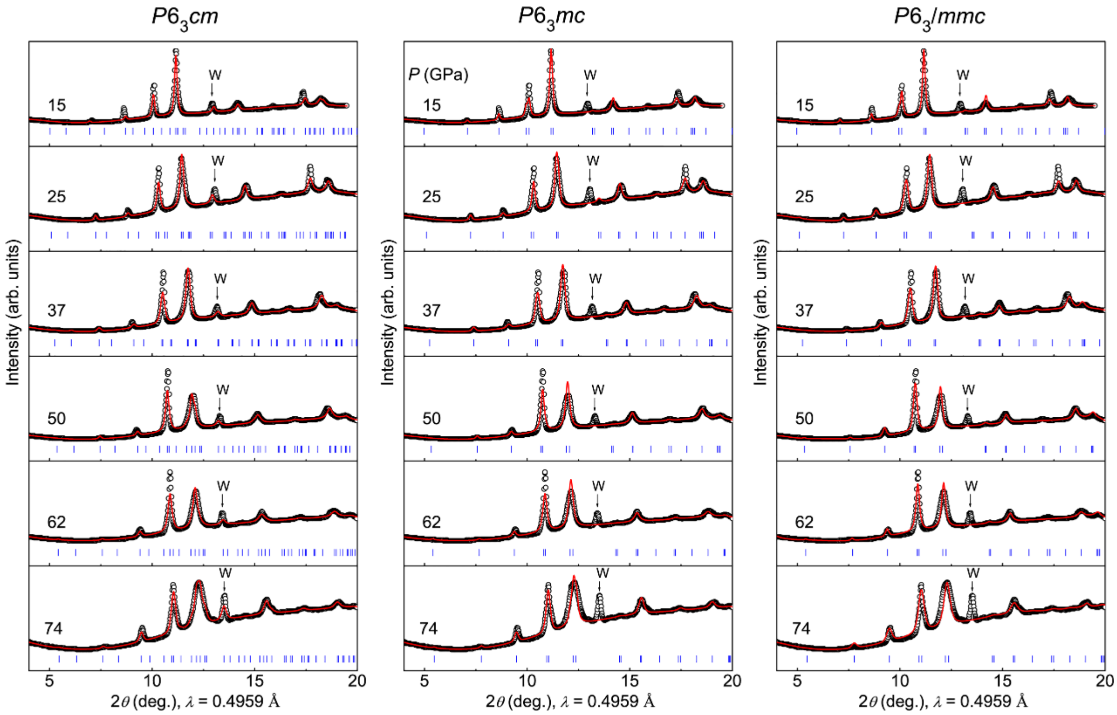

Fig. S11. Rietveld fitting results of the XRD patterns of $BaTiSe_3$ under compression. The open circles and red lines are the experimental data and fitting results using *P*6$_3$*cm*, *P*6$_3$*mc* and *P*6$_3$/*mmc* symmetries. All the XRD patterns can be well indexed into the *P*6$_3$*cm*, *P*6$_3$*mc* and *P*6$_3$/*mmc* hexagonal perovskite structures with quasi-1D TiX_6 octahedral chains. It is beyond the XRD's capability to distinguish the slight differences to further confirm the exact crystal structure. The blue vertical tick marks are the indexed peaks corresponding to the related structure. The arrows mark the peak from the W gasket used in our XRD experiments.

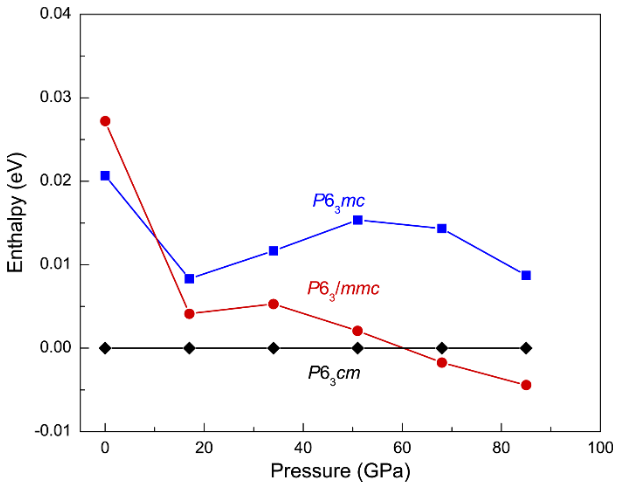

Fig. S12. Enthalpy – pressure curves of the *P*6₃*mc* and *P*6₃/*mmc* structures relative to that of the *P*6₃*cm* structure of $BaTiS_3$. At ambient conditions, enthalpy calculations show that the *P*6₃cm phase is the energetically stable phase, consistent with our recently reported single-crystal XRD results on $BaTiS_3$ (*47*). With application of pressure, the *P*6₃/*mmc* structure becomes more stable and has lower enthalpy compared to the *P*6₃*cm* and *P*6₃*mc* structures above 60 GPa, suggesting a structural transition from *P*6₃*cm* to *P*6₃*mmc*. This potential phase change coincides with the superconducting transition, implying that the superconducting phase of $BaTiS_3$ adopts the *P*6₃/*mmc* symmetry. As mentioned earlier, these structures are very similar, with only slight differences in atomic positions that are beyond the resolution of standard XRD to detect. Therefore, these minor changes were not observed in our high-pressure XRD data. Despite the subtle structural differences, their impact on the electronic structures is minimal (*47*).

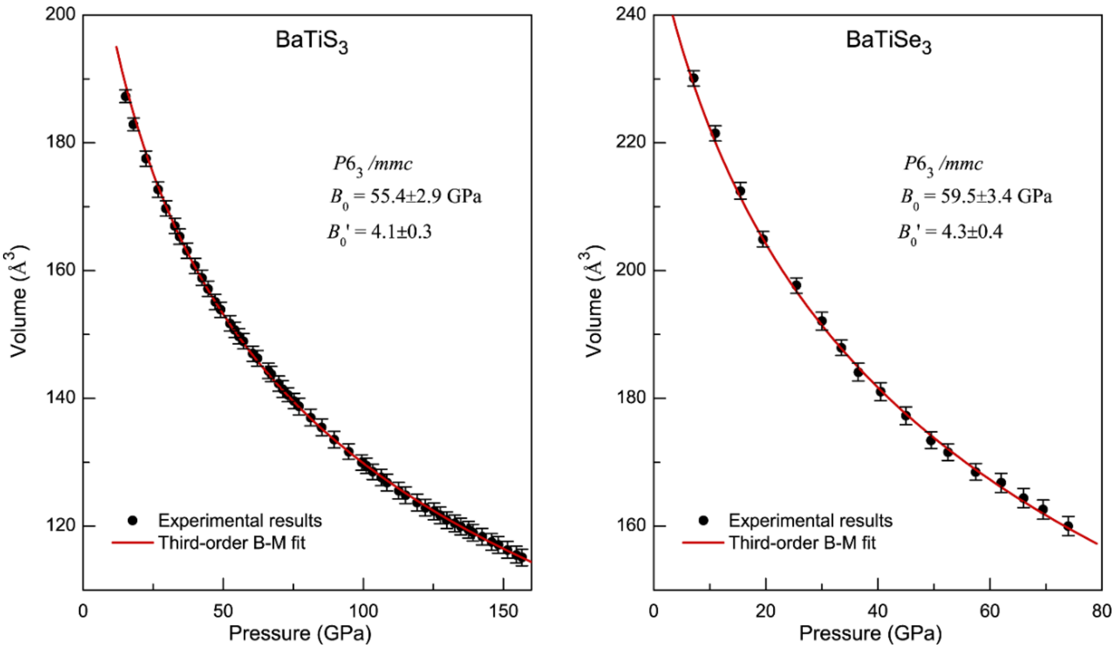

Fig. S13. Pressure dependence of the volume of $BaTiS_3$ and $BaTiSe_3$. Solid circles are the experimental fitting data obtained from the XRD using the $P6_3/mmc$ symmetry, while red lines are the fitting curves based on a third-order Birch-Murnaghan (B-M) equation of state. The volume uncertainty is from the Rietveld refinements of the XRD data. The error bars of bulk moduli arise from both the volume uncertainty and fitting procedure using the third-order B-M equation of state.

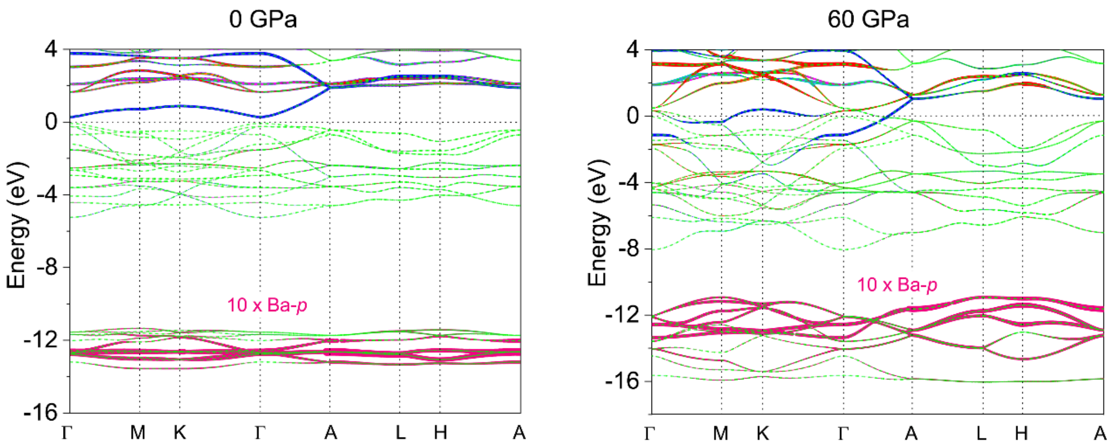

Fig. S14. Band structures of $BaTiS_3$ at 0 and 60 GPa showing the contribution of Ba atoms to the electronic structure. The pink bands around -12 eV are from the *p* orbitals of Ba atoms.

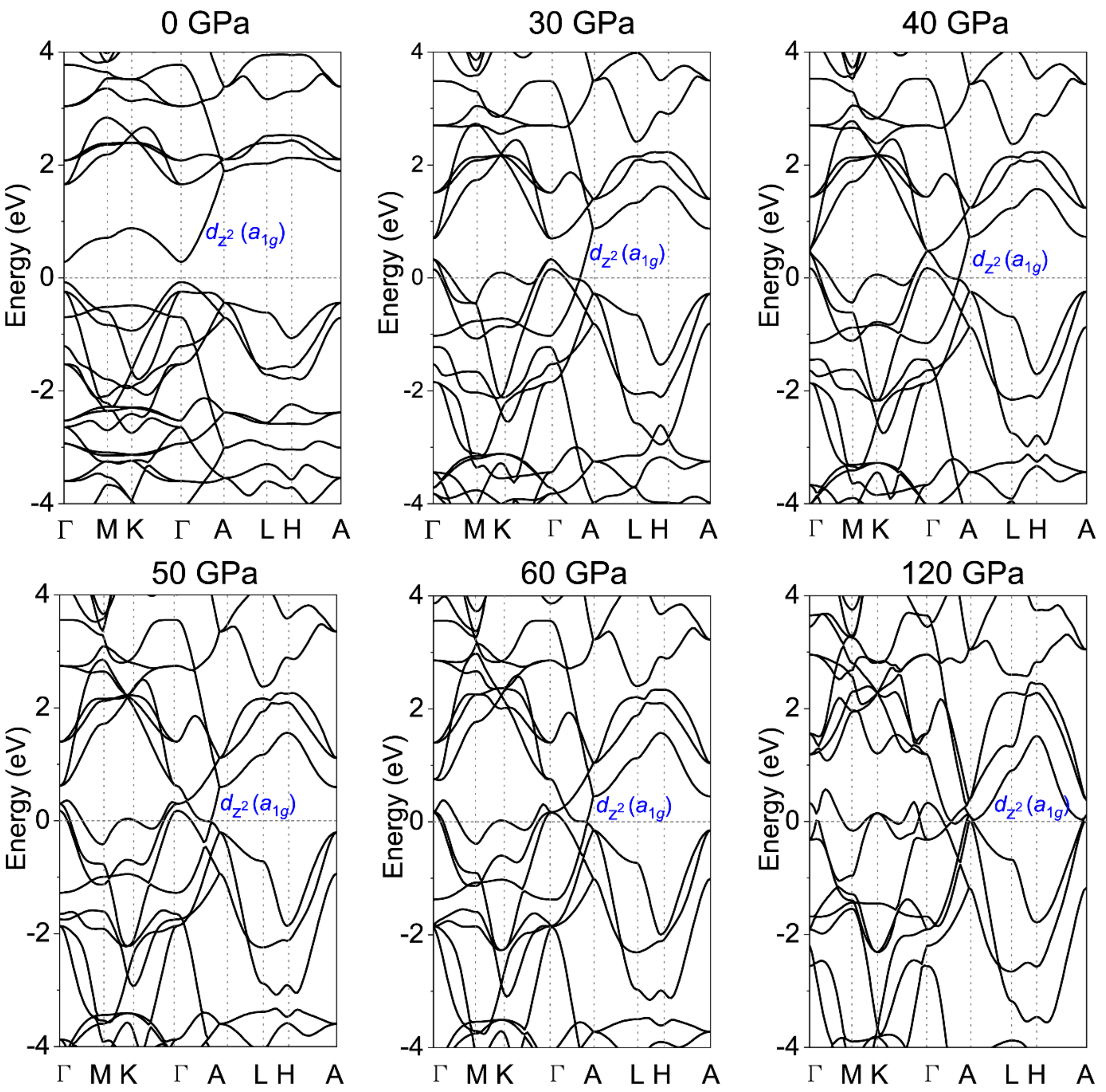

Fig. S15. Band structures of $BaTiS_3$ under compression calculated using the *P*6_3/*mmc* structure.

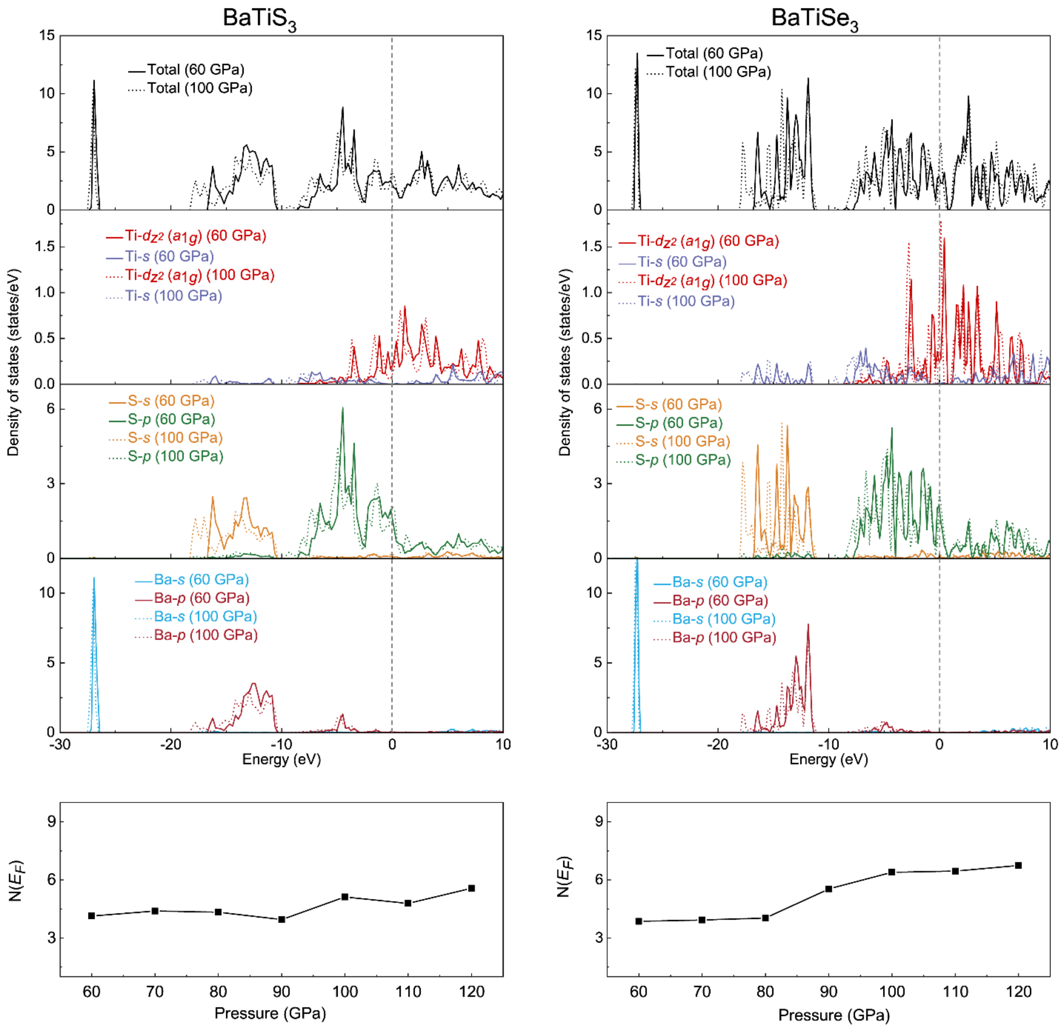

Fig. S16. Density of states (DOS) and their pressure dependence of $BaTiS_3$ (left panels) and $BaTiSe_3$ (right panels) at 60 (solid lines) and 100 GPa (dashed lines). For the Ti-*d* orbitals, we only plot the contribution of Ti-$d_z{}^2$ (a_{1g}) orbital, since it contributes most to the DOS near the Fermi surface. The DOS calculations indicate that $N(E_F)$ of $BaTi(S,Se)_3$ is mainly contributed by the Ti-$d_z{}^2$ (a_{1g}) and S/Se-*p* states. Ba atoms have minimal contributions to the $N(E_F)$. $N(E_F)$ of $BaTiS_3$ is comparable with that of $BaTiSe_3$ at 60 GPa, suggesting that substitution of S with Se has minimal effect on the $N(E_F)$. With further compression, both $N(E_F)$ remain almost unchanged up to 80 GPa, followed by a gentle increase at higher pressures. At 100 GPa, $N(E_F)$ of $BaTiS_3$ is slightly smaller than that of $BaTiSe_3$.

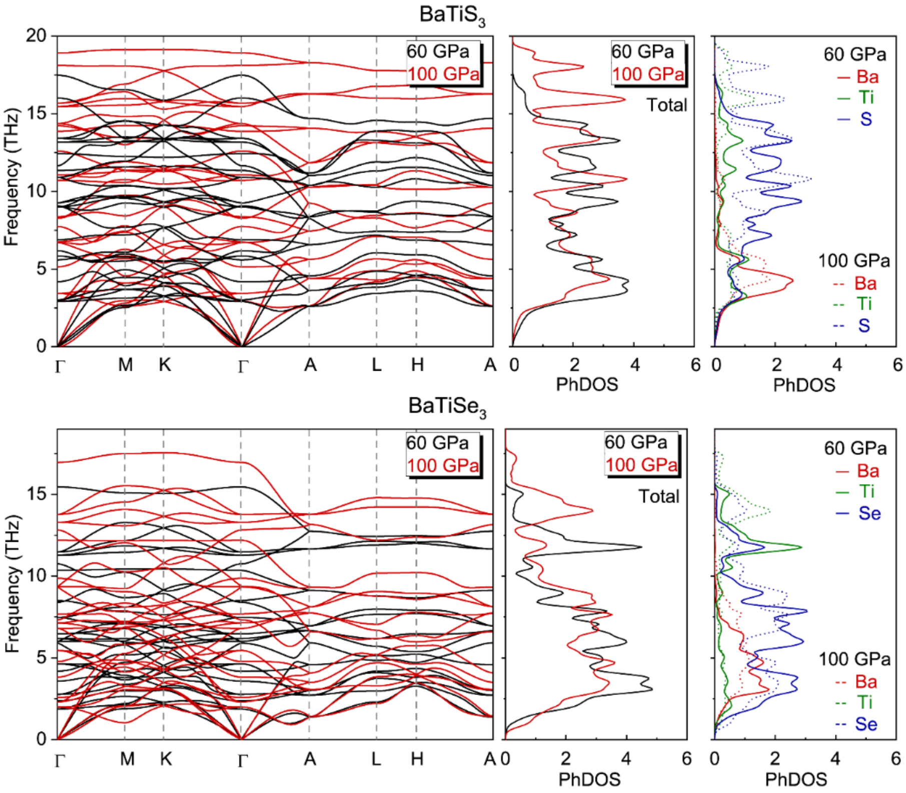

Fig. S17. Phonon dispersion, and total and projected phonon density of states (PhDOS) of $BaTiS_3$ (top panels) and $BaTiSe_3$ (bottom panels) at 60 and 100 GPa calculated using the Vienna ab initio simulation package (VASP). No imaginary phonons are observed in the phonon dispersion patterns, indicating the stability of $P6_3/mmc$ structure under compression, consistent with our XRD results. The PhDOS of $BaTiS_3$ reveal that Ba atoms play a sizable role in the low-frequency phonons below 5 THz, while its contribution to $N(E_F)$ is minimal. Ti and S contribute equally to the low-frequency phonons. In contrast, for $BaTiSe_3$, the low-frequency phonons are mainly from Se atoms, combined with sizable and minimal contributions from Ba and Ti, respectively. The different PhDOS suggest that the phonons paired with electrons to induce superconductivity may be different between $BaTiS_3$ and $BaTiSe_3$.

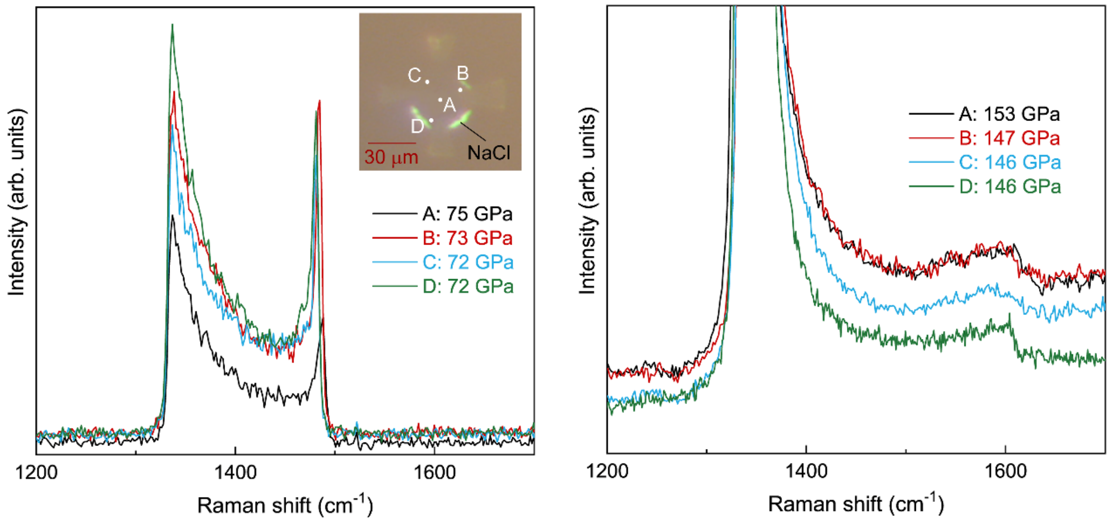

Fig. S18. Pressure determination and pressure gradients for the high-pressure electrical transport measurements using the Raman peak of the diamond anvil. Raman spectra at two representative pressures, ~75 GPa (left) and ~153 GPa (right), are shown, collected from different locations within the sample (inset image). A few GPa (< 5%) difference, for example, ~3 GPa at ~75 GPa and ~7 GPa at ~153 GPa, can be observed between the center and edge of the sample. In this study, pressure values used for data plotting correspond to those measured at the center of the sample.

Table S1. Crystal field splitting of the *d* orbitals in corner-sharing and face-sharing octahedra (*22*). The *z* axis in face-sharing hexagonal perovskite is defined along the metal – metal chain direction.

Corner-sharing octahedral field		Face-sharing octahedral field	
Symmetry	Orbitals	Symmetry	Orbitals
t_{2g}	d_{xy}	a_{1g}	d_{z^2}
	d_{yz}	e_g^{π}	$-\frac{2}{\sqrt{6}}d_{xy}+\frac{2}{\sqrt{3}}d_{yz}$
	d_{xz}		$\frac{2}{\sqrt{6}}d_{x^2-y^2}+\frac{1}{\sqrt{3}}d_{xz}$
e_g	$d_{x^2-y^2}$	e_g^{σ}	$\frac{1}{\sqrt{3}}d_{x^2-y^2}-\sqrt{\frac{2}{3}}d_{xz}$
	d_{z^2}		$-\frac{1}{\sqrt{3}}d_{xy}-\sqrt{\frac{2}{3}}d_{yz}$

Table S2. Comparison between $BaTiX_3$ (X = S, Se) and representative results from previously reported chalcogenide superconductors. References with asterisks indicate observation of pressure-induced superconductivity.

Compounds		Dimensionality	Symmetry	Ref
Hexagonal perovskite chalcogenides	$BaTiS_3$	**Quasi-1D**	*P*6$_3$/*mmc*	This study*
	$BaTiSe_3$	**Quasi-1D**	*P*6$_3$/*mmc*	This study*
PbX (X = S, Se)	$Pb_{0.99}Cr_{0.01}Se$	3D	*Pm*-3*m*	(49)*
	PbSe	3D	*Pm*-3*m*	(50)*
	PbS	3D	*Pm*-3*m*	(51)*
Transition metal chalcogenides	NbS_2	2D	*R*3*m*	(52)
	$NbSe_2$	2D	*P*6$_3$/*mmc*	(52)
	MoS_2	2D	*P*6$_3$/*mmc*	(53)*
	TaS_2	2D	*P*-3*m*1	(54)*
	WS_2	2D	*P*6$_3$/*mmc*	(55)
Iron-based chalcogenides	FeSe	2D	*P*4/*nmm*	(56)
	FeS	2D	*P*4/*nmm*	(57)
	$Fe_{1.01-x}Cu_xSe$	2D	*P*4/*nmm*	(58)*
Others	La_3Se_4	3D	*I*-43*d*	(59)
	La_3S_4	3D	*I*-43*d*	(59)
	$SnNbS_3$	2D/3D	Tetragonal	(60)
	$SnTaS_3$	2D/3D	Tetragonal	(60)
	$SnNbSe_3$	2D/3D	Tetragonal	(60)
	In_2Se_3	3D	*I*-43*d*	(61)*
	SnS	3D	*Pm*-3*m*	(62)*
	SnSe	3D	*Pm*-3*m*	(63)*
	MnSe	3D	*Pnma*	(64)*

Table S3. Structural information of $BaTiS_3$ with $P6_3/mmc$ symmetry at high pressure.

Pressure (GPa)	Lattice parameters (Å)	Atomic coordinates	x	y	z
62 (Experiment)	a = 5.797±0.008	Ba:	0.667	0.333	0.250
	c = 5.025±0.08	Ti:	0.000	0.000	0.500
		S:	0.17(6)	0.80(6)	0.250
60 (Calculation)	a = 5.783	Ba:	0.667	0.333	0.250
	c = 5.095	Ti:	0.000	0.000	0.500
		S:	0.186	0.814	0.250
100 (Experiment)	a = 5.571±0.011	Ba:	0.667	0.333	0.250
	c = 4.844±0.012	Ti:	0.000	0.000	0.500
		S:	0.17(9)	0.80(1)	0.250
100 (Calculation)	a = 5.531	Ba:	0.667	0.333	0.250
	c = 4.901	Ti:	0.000	0.000	0.500
		S:	0.189	0.810	0.250

REFERENCES AND NOTES

1. J. F. Schooley, W. R. Hosler, M. L. Cohen, Superconductivity in semiconducting $SrTiO_3$. *Phys. Rev. Lett.* **12**, 474–475 (1964).

2. A. W. Sleight, J. L. Gillson, P. E. Bierstedt, High-temperature superconductivity in the $BaPb_{1-x}Bi_xO_3$ systems. *Solid State Commun.* **17**, 27–28 (1975).

3. L. F. Mattheiss, E. M. Gyorgy, D. W. Johnson, Jr., Superconductivity above 20 K in the Ba-K-Bi-O system. *Phys. Rev. B* **37**, 3745–3746 (1988).

4. R. J. Cava, B. Batlogg, J. J. Krajewski, R. Farrow, L. W. Rupp, A. E. White, K. Short, W. F. Peck, T. Kometani, Superconductivity near 30 K without copper: The $Ba_{0.6}K_{0.4}BiO_3$ perovskite. *Nature* **332**, 814–816 (1988).

5. M. Kim, G. M. McNally, H.-H. Kim, M. Oudah, A. S. Gibbs, P. Manuel, R. J. Green, R. Sutarto, T. Takayama, A. Yaresko, U. Wedig, M. Isobe, R. K. Kremer, D. A. Bonn, B. Keimer, H. Takagi, Superconductivity in $(Ba,K)SbO_3$. *Nat. Mater.* **21**, 627–633 (2022).

6. D. G. Hinks, D. R. Richards, B. Dabrowski, D. T. Marx, A. W. Mitchell, The oxygen isotope effect in $Ba_{0.625}K_{0.375}BiO_3$. *Nature* **335**, 419–421 (1988).

7. Q. Huang, J. F. Zasadzinski, N. Tralshawala, K. E. Gray, D. G. Hinks, J. L. Peng, R. L. Greene, Tunnelling evidence for predominantly electron–phonon coupling in superconducting $Ba_{1-x}K_xBiO_3$ and $Nd_{2-x}Ce_xCuO_{4-y}$. *Nature* **347**, 369–372 (1990).

8. C. H. P. Wen, H. C. Xu, Q. Yao, R. Peng, X. H. Niu, Q. Y. Chen, Z. T. Liu, D. W. Shen, Q. Song, X. Lou, Y. F. Fang, X. S. Liu, Y. H. Song, Y. J. Jiao, T. F. Duan, H. H. Wen, P. Dudin, G. Kotliar, Z. P. Yin, D. L. Feng, Unveiling the superconducting mechanism of $Ba_{0.51}K_{0.49}BiO_3$. *Phys. Rev. Lett.* **121**, 117002 (2018).

9. Z. Li, G. Antonius, M. Wu, F. H. da Jornada, S. G. Louie, Electron-phonon coupling from ab initio linear-response theory within the *GW* method: Correlation-enhanced interactions and superconductivity in $Ba_{1-x}K_xBiO_3$. *Phys. Rev. Lett.* **122**, 186402 (2019).

10. R. Nourafkan, F. Marsiglio, G. Kotliar, Model of the electron-phonon interaction and optical conductivity of $Ba_{1-x}K_xBiO_3$ superconductors. *Phys. Rev. Lett.* **109**, 017001 (2012).

11. B. Keimer, S. A. Kivelson, M. R. Norman, S. Uchida, J. Zaanen, From quantum matter to high-temperature superconductivity in copper oxides. *Nature* **518**, 179–186 (2015).

12. M. K. Wu, J. R. Ashburn, C. J. Torng, P. H. Hor, R. L. Meng, L. Gao, Z. J. Huang, Y. Q. Wang, C. W. Chu, Superconductivity at 93 K in a new mixed-phase Y-Ba-Cu-O compound system at ambient pressure. *Phys. Rev. Lett.* **58**, 908–910 (1987).

13. Z. Z. Sheng, A. M. Hermann, Bulk superconductivity at 120 K in the Tl–Ca/Ba–Cu–O system. *Nature* **332**, 138–139 (1988).

14. S. S. P. Parkin, V. Y. Lee, E. M. Engler, A. I. Nazzal, T. C. Huang, G. Gorman, R. Savoy, R. Beyers, Bulk superconductivity at 125 K in $Tl_2Ca_2Ba_2Cu_3O_x$. *Phys. Rev. Lett.* **60**, 2539–2542 (1988).

15. A. Schilling, M. Cantoni, J. D. Guo, H. R. Ott, Superconductivity above 130 K in the Hg–Ba–Ca–Cu–O system. *Nature* **363**, 56–58 (1993).

16. L. Gao, Y. Y. Xue, F. Chen, Q. Xiong, R. L. Meng, D. Ramirez, C. W. Chu, J. H. Eggert, H. K. Mao, Superconductivity up to 164 K in $HgBa_2Ca_{m-1}Cu_mO_{2m+2+\delta}$ (m=1, 2, and 3) under quasihydrostatic pressures. *Phys. Rev. B* **50**, 4260–4263 (1994).

17. D. Li, K. Lee, B. Y. Wang, M. Osada, S. Crossley, H. R. Lee, Y. Cui, Y. Hikita, H. Y. Hwang, Superconductivity in an infinite-layer nickelate. *Nature* **572**, 624–627 (2019).

18. H. Sun, M. Huo, X. Hu, J. Li, Z. Liu, Y. Han, L. Tang, Z. Mao, P. Yang, B. Wang, J. Cheng, D.-X. Yao, G.-M. Zhang, M. Wang, Signatures of superconductivity near 80 K in a nickelate under high pressure. *Nature* **621**, 493–498 (2023).

19. Y. Zhu, D. Peng, E. Zhang, B. Pan, X. Chen, L. Chen, H. Ren, F. Liu, Y. Hao, N. Li, Z. Xing, F. Lan, J. Han, J. Wang, D. Jia, H. Wo, Y. Gu, Y. Gu, L. Ji, W. Wang, H. Gou, Y. Shen, T. Ying, X. Chen, W. Yang, H. Cao, C. Zheng, Q. Zeng, J.-g. Guo, J. Zhao, Superconductivity in pressurized trilayer $La_4Ni_3O_{10-\delta}$ single crystals. *Nature* **631**, 531–536 (2024).

20. Z. Chen, Y. Wang, S. N. Rebec, T. Jia, M. Hashimoto, D. Lu, B. Moritz, R. G. Moore, T. P. Devereaux, Z.-X. Shen, Anomalously strong near-neighbor attraction in doped 1D cuprate chains. *Science* **373**, 1235–1239 (2021).

21. D. I. Khomskii, K. I. Kugel, A. O. Sboychakov, S. V. Streltsov, Role of local geometry in the spin and orbital structure of transition metal compounds. *J. Exp. Theor. Phys.* **122**, 484–498 (2016).

22. N. Wagner, R. Seshadri, J. M. Rondinelli, Property control from polyhedral connectivity in ABO_3 oxides. *Phys. Rev. B* **100**, 064101 (2019).

23. I. Ohkubo, T. Mori, d_{z^2} orbital character of polyhedra in complex solid-state transition-metal compounds. *Dalton Trans.* **49**, 431–437 (2020).

24. K. Yamaura, H. W. Zandbergen, K. Abe, R. J. Cava, Synthesis and properties of the structurally one-dimensional cobalt oxide $Ba_{1-x}Sr_xCoO_3$ ($0 \leqslant x \leqslant 0.5$). *J. Solid State Chem.* **146**, 96–102 (1999).

25. K. E. Stitzer, M. D. Smith, J. Darriet, H. C. zur Loye, Crystal growth, structure determination and magnetism of a new hexagonal rhodate: $Ba_9Rh_8O_{24}$. *Chem. Commun.*, 1680–1681 (2001).

26. S. Fop, K. S. McCombie, E. J. Wildman, J. M. S. Skakle, J. T. S. Irvine, P. A. Connor, C. Savaniu, C. Ritter, A. C. McLaughlin, High oxide ion and proton conductivity in a disordered hexagonal perovskite. *Nat. Mater.* **19**, 752–757 (2020).

27. L. T. Nguyen, R. J. Cava, Hexagonal perovskites as quantum materials. *Chem. Rev.* **121**, 2935–2965 (2021).

28. A. P. Petrović, D. Ansermet, D. Chernyshov, M. Hoesch, D. Salloum, P. Gougeon, M. Potel, L. Boeri, C. Panagopoulos, A disorder-enhanced quasi-one-dimensional superconductor. *Nat. Commun.* **7**, 12262 (2016).

29. Z. Y. Liu, Q. X. Dong, P. T. Yang, P. F. Shan, B. S. Wang, J. P. Sun, Z. L. Dun, Y. Uwatoko, G. F. Chen, X. L. Dong, Z. X. Zhao, J.-G. Cheng, Pressure-induced superconductivity up to 9 K in the quasi-one-dimensional KMn_6Bi_5. *Phys. Rev. Lett.* **128**, 187001 (2022).

30. J.-K. Bao, J.-Y. Liu, C.-W. Ma, Z.-H. Meng, Z.-T. Tang, Y.-L. Sun, H.-F. Zhai, H. Jiang, H. Bai, C.-M. Feng, Z.-A. Xu, G.-H. Cao, Superconductivity in quasi-one-dimensional $K_2Cr_3As_3$ with significant electron correlations. *Phys. Rev. X* **5**, 011013 (2015).

31. Z.-T. Tang, J.-K. Bao, Y. Liu, Y.-L. Sun, A. Ablimit, H.-F. Zhai, H. Jiang, C.-M. Feng, Z.-A. Xu, G.-H. Cao, Unconventional superconductivity in quasi-one-dimensional $Rb_2Cr_3As_3$. *Phys. Rev. B* **91**, 020506 (2015).

32. Q.-G. Mu, B.-B. Ruan, K. Zhao, B.-J. Pan, T. Liu, L. Shan, G.-F. Chen, Z.-A. Ren, Superconductivity at 10.4 K in a novel quasi-one-dimensional ternary molybdenum pnictide $K_2Mo_3As_3$. *Sci. Bull.* **63**, 952–956 (2018).

33. J. Zhang, Y. Jia, X. Wang, Z. Li, L. Duan, W. Li, J. Zhao, L. Cao, G. Dai, Z. Deng, S. Zhang, S. Feng, R. Yu, Q. Liu, J. Hu, J. Zhu, C. Jin, A new quasi-one-dimensional compound Ba_3TiTe_5 and superconductivity induced by pressure. *NPG Asia Mater.* **11**, 60 (2019).

34. Z. Y. Liu, J. Li, J. F. Zhang, J. Li, P. T. Yang, S. Zhang, G. F. Chen, Y. Uwatoko, H. X. Yang, Y. Sui, K. Liu, J.-G. Cheng, Quasi-one-dimensional superconductivity in the pressurized charge-density-wave conductor $HfTe_3$. *npj Quantum Mater.* **6**, 90 (2021).

35. C. Pei, W. Shi, Y. Zhao, L. Gao, J. Gao, Y. Li, H. Zhu, Q. Zhang, N. Yu, C. Li, W. Cao, S. A. Medvedev, C. Felser, B. Yan, Z. Liu, Y. Chen, Z. Wang, Y. Qi, Pressure-induced a partial disorder and superconductivity in quasi-one-dimensional Weyl semimetal $(NbSe_4)_2I$. *Mater. Today Phys.* **21**, 100509 (2021).

36. C. An, Y. Zhou, C. Chen, F. Fei, F. Song, C. Park, J. Zhou, H.-G. Rubahn, V. V. Moshchalkov, X. Chen, G. Zhang, Z. Yang, Long-range ordered amorphous atomic chains as building blocks of a superconducting quasi-one-dimensional crystal. *Adv. Mater.* **32**, 2002352 (2020).

37. C. Escribe Filippini, J. Beille, M. Boujida, J. Marcus, C. Schlenker, Pressure effect on the transport properties of superconducting $Li_{0.9}$ Mo_6O_{17} bronze. *Phys. C Supercond. Appl.* **162-164**, 427–428 (1989).

38. J. Z. Ke, C. Dong, H. P. Zhu, W. X. Liu, M. Y. Shi, Y. Q. Du, J. F. Wang, M. Yang, Synthesis and physical properties of the theoretically predicted spin-triplet superconductor $Li_{0.9}Mo_6O_{17}$. *Ceram. Int.* **47**, 25229–25235 (2021).

39. J.-F. Mercure, A. F. Bangura, X. Xu, N. Wakeham, A. Carrington, P. Walmsley, M. Greenblatt, N. E. Hussey, Upper critical magnetic field far above the paramagnetic pair-breaking limit of superconducting one-dimensional $Li_{0.9}Mo_6O_{17}$ single crystals. *Phys. Rev. Lett.* **108**, 187003 (2012).

40. Y.-Y. Sun, M. L. Agiorgousis, P. Zhang, S. Zhang, Chalcogenide perovskites for photovoltaics. *Nano Lett.* **15**, 581–585 (2015).

41. W. Meng, B. Saparov, F. Hong, J. Wang, D. B. Mitzi, Y. Yan, Alloying and defect control within chalcogenide perovskites for optimized photovoltaic application. *Chem. Mater.* **28**, 821–829 (2016).

42. A. Swarnkar, W. J. Mir, R. Chakraborty, M. Jagadeeswararao, T. Sheikh, A. Nag, Are chalcogenide perovskites an emerging class of semiconductors for optoelectronic properties and solar cell? *Chem. Mater.* **31**, 565–575 (2019).

43. B. Sun, S. Niu, R. P. Hermann, J. Moon, N. Shulumba, K. Page, B. Zhao, A. S. Thind, K. Mahalingam, J. Milam-Guerrero, R. Haiges, M. Mecklenburg, B. C. Melot, Y.-D. Jho, B. M. Howe, R. Mishra, A. Alatas, B. Winn, M. E. Manley, J. Ravichandran, A. J. Minnich, High frequency atomic tunneling yields ultralow and glass-like thermal conductivity in chalcogenide single crystals. *Nat. Commun.* **11**, 6039 (2020).

44. S. Niu, G. Joe, H. Zhao, Y. Zhou, T. Orvis, H. Huyan, J. Salman, K. Mahalingam, B. Urwin, J. Wu, Y. Liu, T. E. Tiwald, S. B. Cronin, B. M. Howe, M. Mecklenburg, R. Haiges, D. J. Singh, H. Wang, M. A. Kats, J. Ravichandran, Giant optical anisotropy in a quasi-one-dimensional crystal. *Nat. Photonics* **12**, 392–396 (2018).

45. B. Zhao, M. S. B. Hoque, G. Y. Jung, H. Mei, S. Singh, G. Ren, M. Milich, Q. Zhao, N. Wang, H. Chen, S. Niu, S.-J. Lee, C.-T. Kuo, J.-S. Lee, J. A. Tomko, H. Wang, M. A. Kats, R. Mishra, P. E. Hopkins, J. Ravichandran, Orientation-controlled anisotropy in single crystals of quasi-1D $BaTiS_3$. *Chem. Mater.* **34**, 5680–5689 (2022).

46. F. Yang, K. Li, M. Fan, W. Yao, L. Fu, C. Xiong, S. Jiang, D. Li, M. Xu, C. Chen, G. Zhang, J. Tang, Strongly anisotropic quasi-1D $BaTiS_3$ chalcogenide perovskite for near-infrared polarized photodetection. *Adv Opt Mater* **11**, 2201859 (2022).

47. H. Chen, B. Zhao, J. Mutch, G. Y. Jung, G. Ren, S. Shabani, E. Seewald, S. Niu, J. Wu, N. Wang, M. Surendran, S. Singh, J. Luo, S. Ohtomo, G. Goh, B. C. Chakoumakos, S. J. Teat, B. Melot, H. Wang, A. N. Pasupathy, R. Mishra, J.-H. Chu, J. Ravichandran, Charge density wave order and electronic phase transitions in a dilute d-band semiconductor. *Adv. Mater.* **35**, 2303283 (2023).

48. B. Zhao, H. Mei, Z. Du, S. Singh, T. Chang, J. Li, B. Ilyas, Q. Song, T.-R. Liu, Y.-T. Shao, R. Comin, N. Gedik, N. S. Settineri, S. J. Teat, Y.-S. Chen, S. B. Cronin, M. A. Kats, J. Ravichandran, Infrared optical anisotropy in quasi-1D hexagonal chalcogenide $BaTiSe_3$. *Adv Opt Mater* **12**, 2400327 (2024).

49. L.-C. Chen, P.-Q. Chen, W.-J. Li, Q. Zhang, V. V. Struzhkin, A. F. Goncharov, Z. Ren, X.-J. Chen, Lattice melting and superconductivity in a group IV-VI compound. *Phys. Rev. B* **103**, 214516 (2021).

50. Y. Jiang, C. Pei, Q. Wang, J. Wu, L. Zhang, C. Xiong, Y. Qi, Pressure-induced superconductivity and phase transition in PbSe and PbTe. *Chinese Phys. B* **33**, 126105 (2024).

51. H. Zhang, W. Zhong, Y. Meng, B. Yue, X. Yu, J.-T. Wang, F. Hong, Superconductivity above 12 K with possible multiband features in CsCl-type PbS. *Phys. Rev. B* **107**, 174502 (2023).

52. M. H. Van Maaren, G. M. Schaeffer, Superconductivity in group Va dichalcogenides. *Phys. Lett.* **20**, 131 (1966).

53. Z. Chi, X. Chen, F. Yen, F. Peng, Y. Zhou, J. Zhu, Y. Zhang, X. Liu, C. Lin, S. Chu, Y. Li, J. Zhao, T. Kagayama, Y. Ma, Z. Yang, Superconductivity in pristine $2H_a$–MoS_2 at ultrahigh pressure. *Phys. Rev. Lett.* **120**, 037002 (2018).

54. B. Sipos, A. F. Kusmartseva, A. Akrap, H. Berger, L. Forró, E. Tutiš, From Mott state to superconductivity in 1T-TaS_2. *Nat. Mater.* **7**, 960–965 (2008).

55. S. Jo, D. Costanzo, H. Berger, A. F. Morpurgo, Electrostatically induced superconductivity at the surface of WS_2. *Nano Lett.* **15**, 1197–1202 (2015).

56. F.-C. Hsu, J.-Y. Luo, K.-W. Yeh, T.-K. Chen, T.-W. Huang, P. M. Wu, Y.-C. Lee, Y.-L. Huang, Y.-Y. Chu, D.-C. Yan, M.-K. Wu, Superconductivity in the PbO-type structure α-FeSe. *Proc. Natl. Acad. Sci. U.S.A.* **105**, 14262–14264 (2008).

57. X. Lai, H. Zhang, Y. Wang, X. Wang, X. Zhang, J. Lin, F. Huang, Observation of superconductivity in tetragonal FeS. *J. Am. Chem. Soc.* **137**, 10148–10151 (2015).

58. L. Deng, T. Bontke, R. Dahal, Y. Xie, B. Gao, X. Li, K. Yin, M. Gooch, D. Rolston, T. Chen, Z. Wu, Y. Ma, P. Dai, C.-W. Chu, Pressure-induced high-temperature superconductivity retained without pressure in FeSe single crystals. *Proc. Natl. Acad. Sci. U.S.A.* **118**, e2108938118 (2021).

59. R. M. Bozorth, F. Holtzberg, S. Methfessel, Superconducting lanthanum chalcogenides. *Phys. Rev. Lett.* **14**, 952–953 (1965).

60. M. H. Van Maaren, Superconductivity in tin -group V^a trichalcogenides. *Phys. Lett. A* **40**, 353–354 (1972).

61. F. Ke, H. Dong, Y. Chen, J. Zhang, C. Liu, J. Zhang, Y. Gan, Y. Han, Z. Chen, C. Gao, J. Wen, W. Yang, X.-J. Chen, V. V. Struzhkin, H.-K. Mao, B. Chen, Decompression-driven superconductivity enhancement in In_2Se_3. *Adv. Mater.* **29**, 1701983 (2017).

62. R. Matsumoto, P. Song, S. Adachi, Y. Saito, H. Hara, A. Yamashita, K. Nakamura, S. Yamamoto, H. Tanaka, T. Irifune, H. Takeya, Y. Takano, Pressure-induced superconductivity in tin sulfide. *Phys. Rev. B* **99**, 184502 (2019).

63. X. Chen, P. Lu, X. Wang, Y. Zhou, C. An, Y. Zhou, C. Xian, H. Gao, Z. Guo, C. Park, B. Hou, K. Peng, X. Zhou, J. Sun, Y. Xiong, Z. Yang, D. Xing, Y. Zhang, Topological Dirac line nodes and superconductivity coexist in SnSe at high pressure. *Phys. Rev. B* **96**, 165123 (2017)

64. T. L. Hung, C. H. Huang, L. Z. Deng, M. N. Ou, Y. Y. Chen, M. K. Wu, S. Y. Huyan, C. W. Chu, P. J. Chen, T. K. Lee, Pressure induced superconductivity in MnSe. *Nat. Commun.* **12**, 5436 (2021).

65. G. J. Fischer, Z. Wang, S.-i. Karato, Elasticity of $CaTiO_3$, $SrTiO_3$ and $BaTiO_3$ perovskites up to 3.0 GPa: The effect of crystallographic structure. *Phys. Chem. Miner.* **20**, 97–103 (1993).

66. Z. P. Yin, A. Kutepov, G. Kotliar, Correlation-enhanced electron-phonon coupling: Applications of *GW* and screened hybrid functional to bismuthates, chloronitrides, and other high-T_c superconductors. *Phys. Rev. X* **3**, 021011 (2013).

67. F. Ke, J. Yan, S. Niu, J. Wen, K. Yin, H. Yang, N. R. Wolf, Y.-K. Tzeng, H. I. Karunadasa, Y. S. Lee, W. L. Mao, Y. Lin, Cesium-mediated electron redistribution and electron-electron interaction in high-pressure metallic $CsPbI_3$. *Nat. Commun.* **13**, 7067 (2022).

68. F. Ke, J. Yan, R. Matheu, S. Niu, N. R. Wolf, H. Yang, K. Yin, J. Wen, Y. S. Lee, H. I. Karunadasa, W. L. Mao, Y. Lin, Quasi-one-dimensional metallicity in compressed $CsSnI_3$. *J. Am. Chem. Soc.* **144**, 23595–23602 (2022).

69. Y. Akahama, H. Kawamura, Pressure calibration of diamond anvil Raman gauge to 310 GPa. *J. Appl. Phys.* **100**, 043516 (2006).

70. G. Kresse, J. Furthmüller, Efficient iterative schemes for ab initio total-energy calculations using a plane-wave basis set. *Phys. Rev. B Condens. Matter* **54**, 11169–11186 (1996).

71. J. P. Perdew, K. Burke, M. Ernzerhof, Generalized gradient approximation made simple. *Phys. Rev. Lett.* **77**, 3865–3868 (1996).

72. S. L. Dudarev, G. A. Botton, S. Y. Savrasov, C. J. Humphreys, A. P. Sutton, Electron-energy-loss spectra and the structural stability of nickel oxide: An LSDA+U study. *Phys. Rev. B* **57**, 1505–1509 (1998).

73. L. C. Fu, W. J. Cheng, Y. Liu, L. C. Shi, Y. Peng, J. Zhang, Z. W. Li, X. D. Li, J. L. Zhu, X. C. Wang, C. Q. Jin, Structure and properties of a quasi-one-dimensional compound $BaTiS_3$ under pressure. *High Press. Res.* **44**, 95–104 (2024).

74. S. Klotz, J.-C. Chervin, P. Munsch, G. Le Marchand, Hydrostatic limits of 11 pressure transmitting media. *J. Phys. D Appl. Phys.* **42**, 075413 (2009).
